# Drug-Induced Reduction in 80Q Aggregates in a *Dictyostelium discoideum* Model of PolyQ Disease

**DOI:** 10.3390/biomedicines14091931

**Published:** 2026-08-28

**Authors:** Bindiya Upadhyay, Ansab Akhtar, Ravi Rawat, Mukul Jain

**Affiliations:** 1 Research and Development Cell, Parul University, Vadodara 391760, Gujarat, India; bindiyaupadhyay09@gmail.com; 2 Department of Life Sciences, Parul Institute of Applied Sciences, Parul University, Vadodara 391760, Gujarat, India; 3 Neuroscience Center, School of Medicine, LSU Health, New Orleans, LA 70112, USA; 4 Amity Institute of Pharmacy, Amity University, Sector 125, Noida 201313, Uttar Pradesh, India

**Keywords:** polyglutamine aggregation, autophagy, AMPK signaling, *Dictyostelium discoideum*, proteostasis, neurodegeneration, metabolic modulators

## Abstract

**Background/Objectives**: Polyglutamine (polyQ) expansion induces protein misfolding, aggregation, and neurodegeneration; however, effective strategies for aggregate clearance remain limited. This study evaluated the effect of metformin, resveratrol, and curcumin on polyQ proteotoxicity using an engineered *Dictyostelium discoideum* model expressing 80Q repeats, and investigated their autophagy-related mechanism. **Methods**: In this study, 80Q-expressing *Dictyostelium discoideum* (*D. discoideum*) cells were treated with metformin, resveratrol, or curcumin. Cell viability and proliferation were assessed by growth curve and doubling-time analyses. Developmental assays examined restoration of multicellular morphology. PolyQ aggregation was analyzed using brightfield microscopy, fluorescence microscopy, Thioflavin T (ThT), and Congo Red assays. Quantitative real-time polymerase chain reaction (qRT-PCR) evaluated AMP-activated protein kinase (AMPK) and autophagy-related gene expression. Molecular docking and molecular dynamics (MD) simulations were performed to assess interactions between the compounds and AMPK. **Results**: All three compounds significantly improved viability and reduced doubling time in 80Q-expressing cells. Treated strains displayed improved developmental morphology and culmination compared to untreated controls. Thioflavin T (ThT) and Congo Red assays demonstrated a marked reduction in amyloid-like polyQ aggregates following treatment. Gene-expression analysis revealed the upregulation of AMP-activated protein kinase (AMPK) and core autophagy genes, indicating activation of an AMPK-linked pro-autophagy pathway involved in aggregate clearance. Computational studies further confirmed stable, energetically favorable interactions of metformin, resveratrol, and curcumin with AMPK. **Conclusions**: Metformin, resveratrol, and curcumin effectively reduce polyQ-associated proteotoxicity in *D. discoideum* and are associated with modulation of AMP-activated protein kinase (AMPK) signaling and autophagy-related pathways. These findings support the therapeutic potential of metabolic modulators against protein aggregation and establish *D. discoideum* as a valuable platform for screening autophagy-targeted interventions relevant to neurodegenerative diseases.

## 1. Introduction

Polyglutamine (polyQ) diseases represent a class of inherited neurodegenerative disorders characterized by abnormal expansion of CAG trinucleotide repeats with specific disease-causing genes, leading to the production of toxic protein with extended polyglutamine tracts that form insoluble aggregates, triggering progressive neuronal dysfunction and death [1,2,3]. These include Huntington’s disease several spinocerebellar ataxias, dentatorubral-pallidoluysian atrophy, and spinal and bulbar muscular atrophy, which collectively account for sustainable proportion of monogenic neurodegenerative conditions despite their individual rarity [2,4]. Accumulating evidence indicates that polyQ-protein aggregation is tightly linked to impaired proteostasis disrupted autophagy, mitochondria dysfunction, oxidative stress, and chronic neuroinflammation, all of which converge to accelerate neuronal loss in vulnerable brain regions such as striatum, cortex, and cerebellum [3,5]. The AMP-activated protein kinase (AMPK) signaling pathway has emerged as a central regulator of cellular energy homeostasis, proteostasis, and mitochondrial quality control, making it a compelling therapeutic target in neurodegenerative states [6,7,8]. AMPK acts as an energy-sensing kinase that is activated by decreased ATP/AMP ratios, nutrient deprivations, or pharmacological stimuli, and orchestrates a broad transcriptional and post-translational response that enhanced catabolic process such as autophagy and mitochondrial biogenesis while suppressing anabolic pathways, such as protein and lipid synthesis [7,9].

The activation of AMPK has been shown to promote the clearance of misfolded and aggregated proteins through upregulation of autophagy and chaperone-mediated degradation thereby mitigating proteotoxic stress in several neurodegenerative models [10,11]. In particular, genetic and pharmacological studies in invertebrate and mammalian systems have demonstrated the AMPK activation can ameliorate polyQ-induced toxicity and reduce the burden of polyQ-containing aggregates, suggesting that modulation of this pathway may confer neuroprotection in polyQ disease [4,12]. Among clinically relevant AMPK-activating agents, resveratrol, curcumin, and metformin have attracted considerable attention due to their pleiotropic effects on energy metabolism, oxidative stress, and proteostasis [13,14,15]. Resveratrol, a natural polyphenol found in grapes and berries activates AMPK both directly and indirectly by enhancing the interaction between the upstream kinase LKB1 and AMPK and by stimulating SIRT1-dependent deacetylation events that sensitize the AMPK pathway to energy status signals [13,16]. In preclinical models of neurodegeneration, resveratrol has been shown to enhance the autophagic clearance of amyloid β and other misfolded proteins in a largely AMPK-linked manner, while also improving mitochondrial function and reducing oxidative damage [1,10,17]. Curcumin the principal bioactive compound of turmeric, likewise activates AMPK in multiple tissues, in haptic and neuronal contexts, it increases phosphorylation of AMPK and its downstream target acetyl-Coa carboxylase, leading to downregulation of gluconeogenesis and modulation of autophagy related signaling [15,18,19].

In addition to its AMPK-activating effects, curcumin directly interferes with polyQ protein aggregation by inhibiting components of the endosomal-sorting complex required for transport (ESCRT) machinery, such as Vps36, and by disrupting preformed polyQ-aggregates in yeast and cell-based models [20,21]. Metformin, a widely used biguanide antihyperglycemic drug, mobilizes intracellular AMPK activation through the inhibition of mitochondrial complex-I-dependent ATP production and modulation of the lysosomal v-ATPase network, which, in turn, signals through the LKB1–AMPK axis [22,23]. In neurodegenerative settings, metformin has been reported to enhance autophagic flux and reduce the accumulation of pathogenic proteins, including amyloid-β and mutant huntingtin, in cultured neurons and animal models [1,24,25,26]. A synergistic low-dose combination of metformin and other AMPK-activating agents have demonstrated enhanced suppression of polyQ aggregation and amelioration of polyQ-induced toxicity in *Caenorhabditis elegans*, suggesting that mild, targeted AMPK activation may be more beneficial than intense or chronic stimulation, which can be paradoxically impair synaptic and neuronal health markers [4,25]. These findings support the hypothesis that coordinated AMPK activation by resveratrol, curcumin, and metformin may converge on overlapping mechanisms, including autophagy induction, mitochondrial remodeling, and attenuation of oxidative stress, to counteract polyQ-protein aggregation and protect neurons from degeneration [10,27,28,29]. The key challenge remains translating these observations into safe and effective therapies for polyQ-disease. AMPK signaling is highly context-dependent, and both insufficient and excessive activation can disrupt metabolic homeostasis, synaptic plasticity, and neuroinflammatory responses [6,30]. Furthermore, important pharmacokinetic limitations, such as low brain bioavailability of curcumin, dose-dependent side effects of metformin, and variable tissue-specific response to resveratrol, must be carefully addressed in the design of preclinical and clinical studies [14,18,22]. Emerging research on targeted autophagy modulators, nanomedicine-based delivery systems, and combination strategies that fine-tune AMPK activity without inducing global energy stress offers promising avenues for optimizing neuroprotective regimens in polyQ-disorders [31,32,33]. Within this framework, the present work aimed to investigate the effects of resveratrol, curcumin, and metformin on polyQ-induced proteotoxicity and aggregation in a *Dictyostelium discoideum* model expressing expanded polyglutamine repeats. Specifically, we evaluated their impact on cellular growth, developmental phenotypes, aggregate burden, and the expression of AMPK and autophagy-related genes, with the goal of identifying the pathways associated with aggregate clearance and cellular protection.

## 2. Materials and Methods

### 2.1. Molecular Docking

#### 2.1.1. Protein–Protein Docking of the AMPK–ULK1 Complex

The three-dimensional structures of human AMP-activated protein kinase (AMPK; PDB ID: 4CFF) and human ULK1 (PDB ID: 8SOI) were retrieved from the RCSB Protein Data Bank and prepared using the Protein Preparation Wizard in Maestro (Schrödinger Suite 2021-2) prior to docking. Both protein structures were derived from *Homo sapiens*.

Protein–protein docking studies were performed using the Biologics Module of Schrödinger Suite 2021-2. In the docking protocol, ULK1 was designated as the ligand protein, whereas AMPK was selected as the receptor protein. Prior to docking, both protein structures were prepared using the Protein Preparation Wizard in Maestro.

During the docking setup, the number of ligand rotations to probe was set to 70,000, and the maximum number of docking poses to be generated was limited to 30. The docking procedure was carried out using the default parameters of the Biologics Module to predict the most favorable interaction conformations between AMPK and ULK1 proteins.

#### 2.1.2. Selection of the Representative AMPK–ULK1 Complex

Among the generated docking poses, the protein–protein complex exhibiting the best docking score and favorable intermolecular interactions was selected as the representative AMPK–ULK1 complex. The selected docking frame, provided in the supplementary dataset, was used for subsequent ligand docking, molecular dynamics simulation, and further computational analyses [34,35].

### 2.2. Receptor Grid Generation

Following protein preparation, a receptor grid was generated around the active site with a grid box size of 20 Å × 20 Å × 20 Å. The co-crystallized inhibitor, Resveratrol-AMPK–ULK1 complex, present with the protein structure, was used as the centroid to define the binding site for grid generation.

### 2.3. Ligand Preparation

The structures of all test compounds [Metformin, Resveratrol, Curcumin] and the co-crystallized inhibitor were built using the 2D sketcher in Maestro. Ligand preparation was performed using the LigPrep module, which generated possible ionization states at pH 7.0 ± 2.0 using Epik, tautomeric forms, stereoisomers, and ring conformations. Energy minimization and 3D optimization were carried out using the OPLS3 force field. All possible conformers were generated using the ConfGen module with default settings (a maximum of 32 conformers per ligand). The prepared ligand library was saved in .maegz format for docking.

### 2.4. Molecular Docking Protocol

Molecular docking was performed using the Glide module of Schrödinger Suite 2021 (Schrödinger, LLC, New York, NY, USA) in Standard Precision (SP) mode to evaluate protein–ligand interactions and binding affinities. The computational studies were conducted on a workstation equipped with an Intel Core i7 processor (3.40 GHz × 8) and 16 GB of RAM, running Ubuntu 24.04.4 LTS. Epik state penalties were added to the docking score, and planarity of conjugated π-systems was enhanced during docking calculations [36]. To validate the docking protocol, the co-crystallized inhibitor was extracted from the binding site, re-prepared, and re-docked into the generated receptor grid.

### 2.5. Molecular Dynamics Simulation

Molecular dynamics (MD) simulation was carried out using GROMACS 2025.3.

*(a)*  *Preparation of the enzyme*

The three-dimensional (3D) models of ligand–protein complexes were exported to .pdb format using PyMOL v3.1.0 [37,38,39]. Protein topology was constructed using pdb2gmx with the CHARMM27 force field [40], and ligand topology was generated using ACPYPE [41].

*(b)*  *Setting up the system for simulation*

After applying the force field, the complexes were solvated using the TIP3P water model in a cubic simulation box with periodic boundary conditions, maintaining a minimum distance of 1 nm between the protein surface and the box edge. The system was neutralized by adding appropriate Na^+^ and Cl^−^ ions. Energy minimization was carried out for 50,000 steps using the steepest descent algorithm to remove steric clashes and unfavorable contacts.

Following minimization, the system was equilibrated by performing a 100 ps NVT (constant number of particles, volume, and temperature) simulation at 300 K, followed by a 100 ps NPT (constant number of particles, pressure, and temperature) simulation at 1 bar pressure. Temperature coupling was maintained using the V-rescale thermostat, while pressure coupling was controlled using the Parrinello–Rahman barostat. The Leapfrog integrator was employed throughout the simulations.

Bond lengths were constrained using the LINCS algorithm. Neighbor searching and van der Waals interactions were calculated using the Verlet cutoff scheme. Electrostatic interactions were treated using the Particle Mesh Ewald (PME) method, with Coulomb and van der Waals cutoffs set to 1.2 nm. Finally, a 100 ns molecular dynamics (MD) simulation was performed under isothermal–isobaric (NPT) conditions at 300 K and 1 bar using GROMACS to evaluate the stability and dynamic behavior of the AMPK–ULK1 complexes [42].

*(c)*  *Visualization and analysis of simulation*

The trajectory files were visualized using HeroMDAnalysis [43] and analyzed using XMGrace 5.1.25 [44].

### 2.6. Cell Culture and Assays in D. discoideum

#### 2.6.1. Cell Culture and Maintenance

eYFP cells were used for all in vitro studies. Ax2 cells were grown and maintained in HL5 medium in Petri dishes at 22 °C. For large-scale cultures, cells were inoculated from Petri plates into flasks containing fresh HL5 medium and grown under shaking conditions (120 rpm, 22 °C) until the log phase (3–5 × 10^6^ cells mL^−1^) was reached.

#### 2.6.2. Cell Proliferation Assay

Logarithmic–phasic eYFP cells (2.5–5 × 10^6^ cells mL^−1^), prepared from fresh spores, were diluted into fresh HL5 medium at ~3–5 × 10^5^ cells mL^−1^. Cell density was counted every 12 h up to 96 h using a hemocytometer under a Lambomed TCM 400 microscope (Labomed, Gurgaon, Haryana, India). Proliferation was plotted as cell number versus time. *Doubling time was calculated using the formula given below:*

*Td* = (*t2* − *t1*) × *log2*/*log* (*q2*/*q1*)

*where*

*t1: initial time*

*t2: final time*

*q1: growth at t1*

*q2: growth at t2.*

#### 2.6.3. Electroporation and Transformation of *Dictyostelium discoideum*

The transformation of *D. discoideum* cells was performed by electroporation following the method described by Lynne Pang and David Knecht with minor modifications. Approximately 5 × 10^6^ cells were harvested by centrifugation at 1500 rpm for 5 min at 4 °C and washed twice with ice-cold H-50 buffer (20 mM HEPES, 50 mM KCl, 10 mM NaCl, 1 mM MgSO_4_, 5 mM NaHCO_3_, and 1 mM NaH_2_PO_4_; pH 7.0). The cell pellet was resuspended in 100 μL of ice-cold H-50 buffer, and 10 μg of plasmid DNA was added to the suspension. The mixture was transferred into a pre-chilled 0.1 cm gap electroporation cuvette and electroporated twice at 0.75 kV and 25 μF, with an interval of approximately 5 s between pulses. Following electroporation, cells were incubated on ice for 5 min and then transferred to 10 m Petri dishes containing 10 mL of HL-5 medium for recovery. Cells were allowed to recover overnight before the addition of the appropriate selective antibiotic. Stable transformants were selected using G418 (10 μg/mL), depending on the resistance marker encoded by the expression construct.

#### 2.6.4. Cell Viability Assay

Metformin, resveratrol, and curcumin (analytical grade) were used in this study. Stock solutions (100 mM) were prepared by dissolving metformin in distilled water, whereas resveratrol and curcumin were dissolved in dimethyl sulfoxide (DMSO). Working concentrations were freshly prepared in HL5 medium before each experiment. IC_50_ values were determined by cell number-based dose–response experiments using the following concentration ranges: metformin (5, 7.5, and 10 µM), curcumin (25, 45, and 65 µM), and resveratrol (25, 50, 75, and 100 µM). From these dose-response assays, representative working concentrations were selected from the linear portion of the curves: 10 µM metformin, 45 µM curcumin, and 100 µM resveratrol. Vehicle control (DMSO < 0.1%) and untreated control groups were included in all experiments. Log-phase eyfp cells at a density of approximately 3–5 × 10^5^ cells mL^−1^ were exposed to the indicated compounds or corresponding controls and incubated for 48 h at 22 °C under shaking conditions (120 rpm). After incubation, cell viability was determined by hemocytometer counting in duplicate using a Lambomed TCM 400 microscope and expressed as a percentage relative to the untreated control.

#### 2.6.5. Development Assay

Log-phase cells were harvested, washed twice in chilled 1× KK_2_ buffer (10 mM potassium phosphate, pH 6.5), and resuspended at 5 × 10^7^ cells mL^−1^. Aliquots (10/20 µL) were plated on 1.5% non-nutrient agar plates. Development was synchronised by incubating plates at 4 °C for 4–6 h, then transferring to 22 °C. Images of developing structures were captured every 4 h using an Olympus SZ×7 stereomicroscope (Olympus, Tokyo, Japan).

#### 2.6.6. Congo Red and Thioflavin T (ThT) Assays

In the Congo Red absorbance assay, the dye (50 mM) was prepared in a buffer (5 mM potassium phosphate, 150 mM NaCl, pH 7.4) and filtered through a 0.2 µm syringe. Congo Red dye (190 µL) was incubated with 20 µg of protein samples for 1 h at 37 °C. Absorbance spectrum was recorded from 400–600 nm on a Multimode ELISA microplate reader (Agilent BioTek, Synergy H1, Winooski, VT, USA) [45].

In the ThT binding assay, the dye (5 mM) was prepared in glycine-NaOH buffer and filtered through a 0.2 µm syringe. From this stock solution, 50 µM was prepared and incubated with 10 µg of protein samples for 15 min at 25 °C in the dark. Hence, the resulting ThT fluorescence intensities in the samples were measured at an emission wavelength of 480 nm and an excitation wavelength of 440 nm using a BioTek Synergy H1 microplate reader (Agilent BioTek, Winooski, VT) [46].

#### 2.6.7. Cell Flattening and Fixation for Microscopy

For all flattening and imaging procedures, logarithmic-phase *D. discoideum* eYFP cells were harvested, washed twice with chilled 1× KK_2_ buffer, and resuspended in KK_2_ buffer. For cell flattening using the agar overlay technique, acid-washed glass coverslips were placed in a sterile Petri dish and overlaid with a thin layer (1–2 mm) of 1% low-melting-point agarose in phosphate-buffered saline (PBS). A small aliquot of the cell suspension in KK_2_ buffer was placed beneath the agar layer and allowed to attach for 10 min at room temperature. The agar layer was carefully removed, and the cells were immediately fixed. Cells were fixed with freshly prepared fixative solution containing 3.7% formaldehyde, 0.05% glutaraldehyde, and 0.1% Triton X-100 in 50% PHEM buffer (pH 6.9) for 15 min at room temperature. After fixation, the coverslips were washed three times with PBS and prepared for confocal imaging [47].

All *D. discoideum* culture and assay procedures were performed according to the DictyBase protocol (http://dictybase.org/).

#### 2.6.8. Western Blot Analysis

Protein expression levels of AMPK and phosphorylated AMPKα (Thr172) were determined by SDS–polyacrylamide gel electrophoresis (SDS-PAGE) followed by Western blotting. Total cellular proteins were extracted and quantified using standard procedures. Equal amounts of protein (40 µg) were separated on a 6% SDS-polyacrylamide gel according to the method described by [48]. The resolved proteins were transferred onto a PVDF membrane and blocked with 5% non-fat skim milk in pBST for 1 h at room temperature. Membranes were then incubated overnight at 4 °C with primary antibodies against AMPKα and phospho-AMPKα (Thr172) (Cell Signalling Technology, Danvers, MA, USA) at a dilution of 1:1000. Following washing with PBST, membranes were incubated with secondary antibodies for 1 h at 4 °C. Protein bands were visualized using an enhanced chemiluminescence (ECL) detection system and quantified by densitometric analysis. β-Actin was used as the loading control for the normalization of protein expression levels [48].

### 2.7. Gene Set Selection

The STRING database (https://string-db.org/) contains over fifty-two million macromolecules from more than 1100 species, primarily used to establish protein–protein interactions (PPIs). To analyze the 4 selected targets, they were entered into STRING. The PPI network was retrieved and loaded into Cytoscape 3.7.1 (https://cytoscape.org/download.html) accessed on 3 March 2026. Topological analysis was performed using the criterion that node degree exceeds the median to screen for significant targets [49].

### 2.8. Statistical Analysis

Data collection and summarization were performed using Microsoft Excel; all experiments were conducted independently at least three times (n = 3), with each experiment performed in triplicate. Data are presented as the mean ± SD. A one-way analysis of variance was used to compare the groups. Statistical analysis was performed using GraphPad Prism 8.0 (GraphPad Software, San Diego, CA, USA) and ImageJ Version 1.54p (https://imagej.net/ij/download.html) accessed on 22 March 2024. A *p*-value < 0.05 was considered statistically significant, with significance indicated as *p* < 0.05, *p* < 0.01, and *p* < 0.001. All images were captured at a resolution corresponding to a scale bar of 100 µm.

## 3. Results

### 3.1. Molecular Dynamics Analysis Reveals Stable Binding and Favorable Energetics of Metformin with Human AMPK

**Figure 1:**
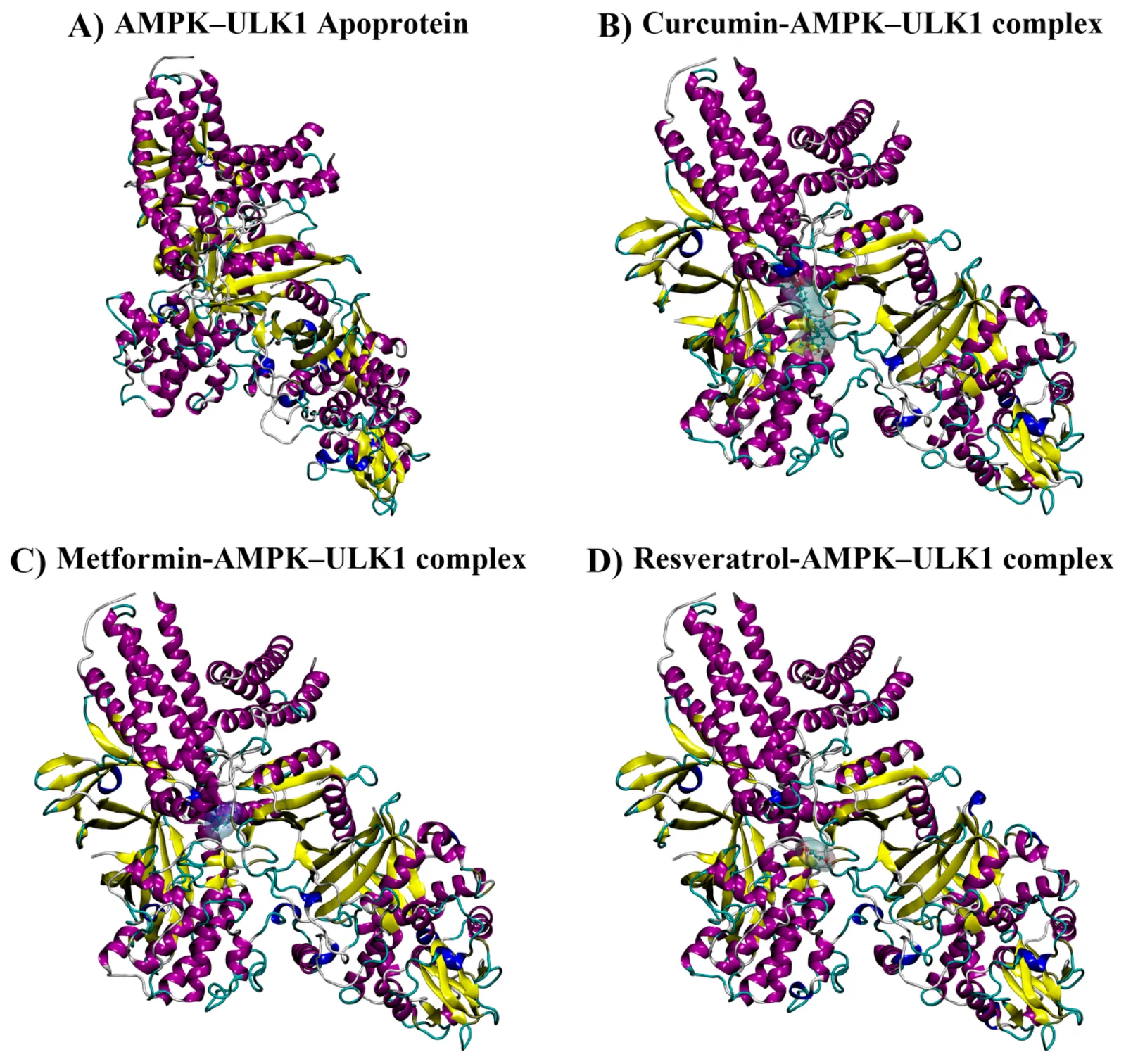
Molecular dynamics simulations analysis of AMPK–ULK1 apoprotein and ligand-bound complexes. Representative structural snapshot of (**A**) AMPK–ULK1 apoprotin, (**B**) curcumin–AMPK–ULK1 complex, (**C**) metformin–AMPK–ULK1 complex, and (**D**) resveratrol–AMPK–ULK1 complex. The ligand-bound systems exhibited overall structural stability, with minor conformational adjustments, indicating sustained protein integrity and favorable ligand accommodation throughout the simulation.

The 3D structure of the metformin–AMPK complex (Supplementary Figure S1A) demonstrates that metformin forms a stable, energetically favorable complex within a defined kinase pocket, maintaining consistent binding geometry with minimal steric clashes and anchored by persistent hydrogen bonds. Protein and ligand RMSD analyses (Supplementary Figure S1B) confirm conformational stability over 100 ns, with the backbone plateauing in a narrow range and the ligand RMSD remaining low, indicating no major rearrangements or dissociation. Local flexibility (Supplementary Figure S1C) is modest, with per-residue RMSF showing only minor peaks at the binding cavity, supporting a well-tolerated, non-disruptive binding mode compatible with enzymatic regulation. The hydrogen bonding network (Supplementary Figure S1D) persists across the trajectory with brief interruptions, reflecting specific recognition rather than transient contacts and contributing to the observed low ligand RMSD. MM/GBSA binding free energies (Supplementary Figure S1E) remain consistently negative (−40 to −55 kcal·mol^−1^; average ΔG ≈ −49.6 kcal·mol^−1^), confirming thermodynamic favorability without affinity loss. Docking of the AMPK–ULK1 complex yielded a top PIPER score of −146.579 (Supplementary Table S1), and MD analysis confirmed stable ligand-bound complexes with RMSD < 0.5 nm and persistent hydrogen bonds. MM/GBSA further supported favorable binding for curcumin (−49.63 kcal·mol^−1^) and resveratrol (−46.13 kcal·mol^−1^), consistent with prior data. Figure 1 shows that ligand-bound AMPK–ULK1 complexes retain compact tertiary structure with subtle adaptive rearrangements, indicating that ligand binding stabilizes key conformational regions without perturbing the protein fold, supporting their potential to modulate AMPK–ULK1 activity through persistent, energetically preferred interactions.

#### 3.1.1. RMSD Analysis

**Figure 2:**
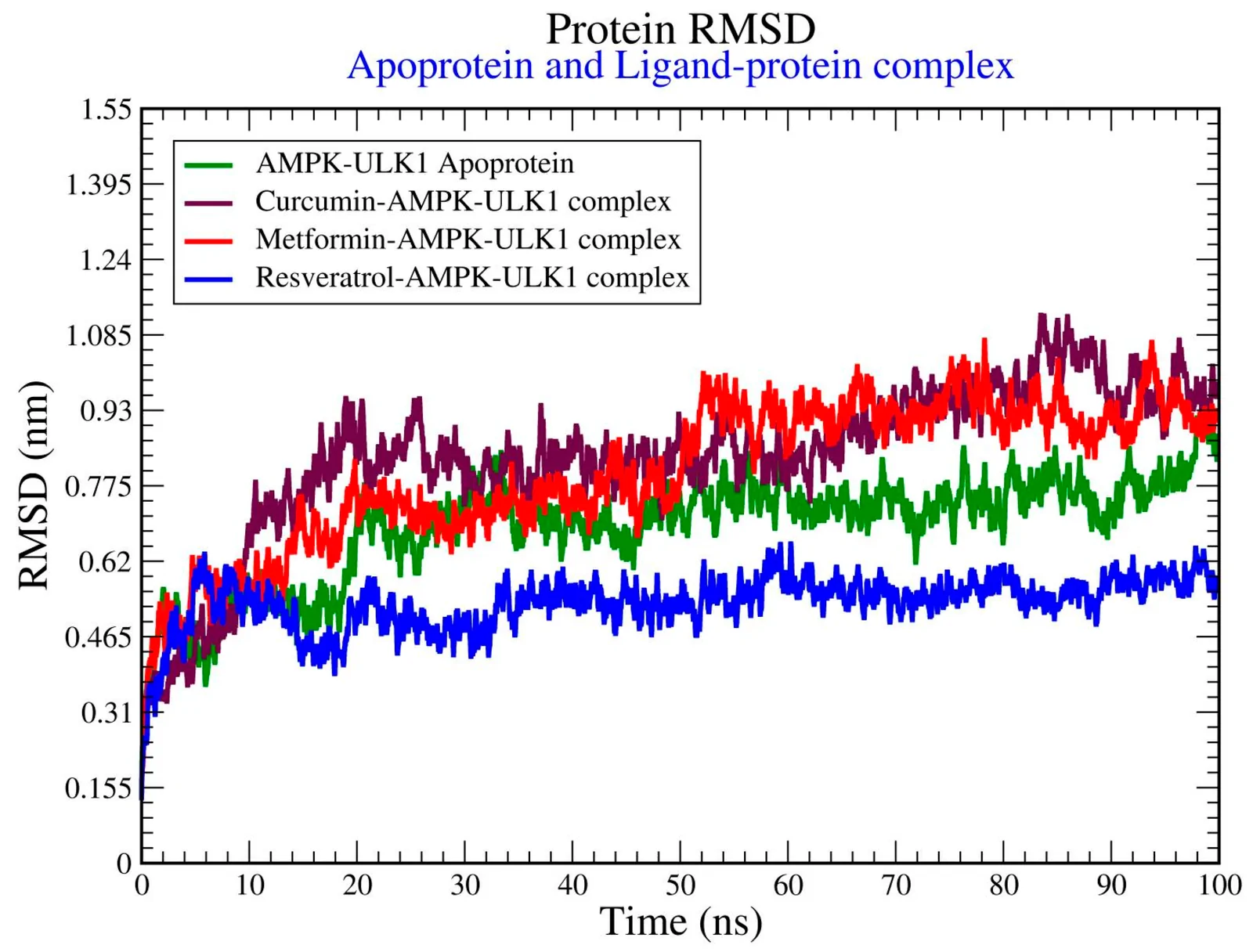
RMSD analysis of the AMPK–ULK1 apoprotin and ligand-bound complexes over 100 ns of molecular dynamics simulations. The apoprotin (green) and the complexes with curcumin (purple), metformin (red) and resveratrol (blue) were monitored to assess structural stability and conformation changes. The resveratrol AMPK–ULK1 complex displayed the lowest and most stable RMSD value, whereas the curcumin and metformin complexes showed higher fluctuations, indicating greater conformational adjustment during the simulation.

The RMSD plot indicates the overall structure stability and conformation behavior of the AMPK–ULK1 apoprotin and its ligand bound complexes during the 100 ns simulation. The apoprotin shows moderate fluctuation and gradually stabilizes around 0.7–0.8 nm, suggesting intrinsic flexibility of the unbound protein. Among the ligand bound systems, the resveratrol complex exhibits the lowest RMSD value throughout the simulation, remaining relatively stable at 0.45–0.6 nm, suggesting that resveratrol may impart greater conformational stability to the AMPK–ULK1 Complex. In contrast, the curcumin and metformin complexes show higher RMSD value, with noticeable fluctuations and gradual increase towards 0.9–1.0 nm, indicating more pronounced structural rearrangement but still within a range consistent with a stable protein ligand complex. Overall, the RMSD profile suggests that all ligands are accommodated by the Protein while resveratrol appears to maintain the most stable conformational state among the tested compounds (Figure 2). The ligand RMSD profiles show the stability of each ligand within the AMPK–ULK1 binding pocket over the 100 ns simulation. All three ligands remain within relatively low RMSD ranges, indicating that they stayed bound without major displacement from the initial docking pose. Resveratrol shows the most stable behavior overall, with lower and more consistent fluctuations, suggesting a stronger and more persistent fit in the binding site. Metformin displays moderate fluctuation but remains largely stable throughout the trajectory, while curcumin shows slightly higher oscillations during the middle portion of the simulation, reflecting temporary repositioning or adaptive adjustment within the pocket. Overall, these results indicated that all three ligands maintain favorable binding stability, with Resveratrol appearing to be the most conformationally stable ligand in the AMPK–ULK1 complex (Supplementary Figure S2). RMSF analysis revealed that ligand binding reduced residue fluctuations of the AMPK–ULK1 complex compared to the apo protein during the 100 ns simulation. Among all compounds, the resveratrol-bound complex exhibited the lowest RMSF values, indicating the highest structural stability and reduced conformational flexibility (Supplementary Figure S3).

#### 3.1.2. Hydrogen Bond Interaction Analysis

**Figure 3:**
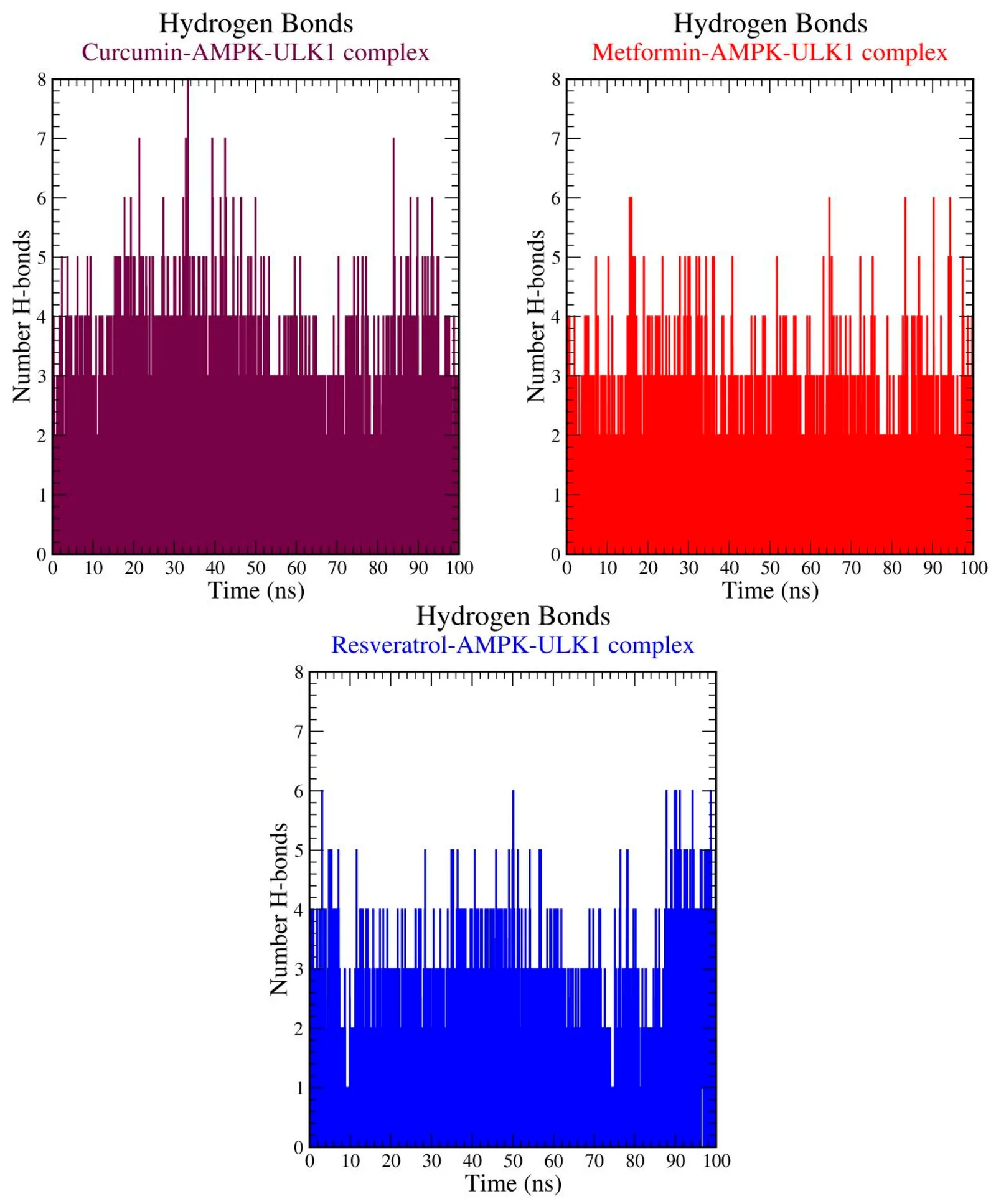
Number of hydrogen bonds between ligands and the Ulk 1 protein over a 100 ns simulation trajectory. The plots show the variation in the number of hydrogen bond for the curcumin metformin and resveratrol complexes. The frequent persistence of these interactions throughout the simulation period indicates stable binding each ligand within the AMPK–ULK1 complex binding pocket.

The hydrogen bond analysis reveals the persistence and frequency of intermolecular interactions between the ligand and the AMPK–ULK1 protein throughout the 100 ns simulation. All three complexes demonstrated stable hydrogen bonding with the majority of the simulation time showing at least 2–4 hydrogen bond, confirming the sustained binding affinity curcumin metformin and resveratrol within active site. Curcumin exhibits the highest peak in hydrogen bond count (up to 8), suggesting a robust, potentially multifaceted interaction network while metformin and resveratrol maintain a consistently high average frequency of 2–3 bonds these results collectively support the stability of the docked configuration, indicating that these ligands from reliable persistent interactions with the target protein, necessary for effective biological modulation (Figure 3). Hydrogen bond occupancy analysis revealed distinct interaction patterns among the three ligand–AMPK–ULK1 complexes. In the metformin–AMPK–ULK1 complex, the most prominent interactions were observed with TRP894 (19.70%) and GLU893 (18.34%), followed by LEU957 (10.12%), indicating stable but moderately distributed hydrogen-bond interactions. In the Resveratrol–AMPK–ULK1 complex, LEU206 (28.88%) and VAL460 (21.85%) exhibited the highest occupancies, suggesting strong and persistent binding interactions that contribute to complex stability. In contrast, the curcumin–AMPK–ULK1 complex demonstrated the strongest hydrogen-bonding network, with ASP461 showing the highest occupancy (36.83%), followed by VAL460 (16.52%), PRO208 (14.26%), ARG966 (13.36%), and LEU206 (11.44%). Overall, curcumin exhibited the most persistent hydrogen-bond interactions, followed by resveratrol, while metformin displayed comparatively lower occupancy values, indicating that curcumin and resveratrol may form more stable complexes with AMPK–ULK1 during the molecular dynamics simulation (Supplementary Figure S4).

#### 3.1.3. MM-PBSA Calculations

MM-PBSA analysis (Supplementary Figures S5 and S6, and Supplementary Table S2) shows negative total binding free energies for all systems (ΔG = −27.15 ± 3.11 kcal·mol^−1^ for apo AMPK–ULK1; −15.50 ± 2.51 for curcumin; −15.56 ± 4.79 for metformin), indicating spontaneous, thermodynamically stable binding driven primarily by van der Waals interactions (−34.55, −19.91, and −19.55 kcal·mol^−1^, respectively) that overcome the positive polar solvation penalty (19.98, 10.28, and 10.53 kcal·mol^−1^). The ΔG time traces remain consistently negative over the 100 ns trajectory (Supplementary Figure S5), and mean ΔG values confirm comparable affinities for curcumin and metformin (Supplementary Figure S6B). These results support stable, persistent ligand–AMPK–ULK1 interactions and suggest a potential involvement of AMPK-associated autophagy pathways in the observed cellular responses.

#### 3.1.4. Principal Component and Free Energy Landscape Analysis

**Figure 4:**
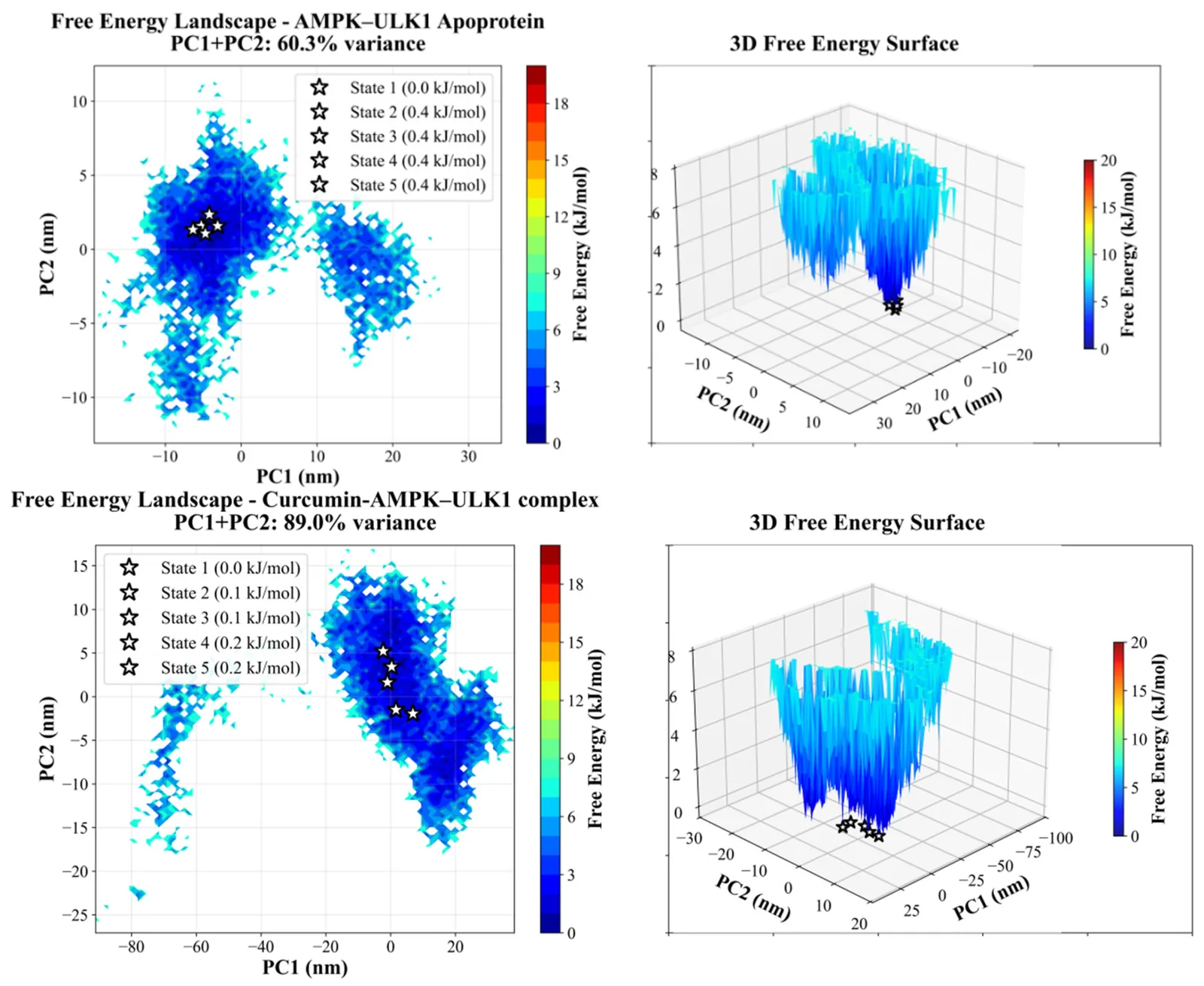

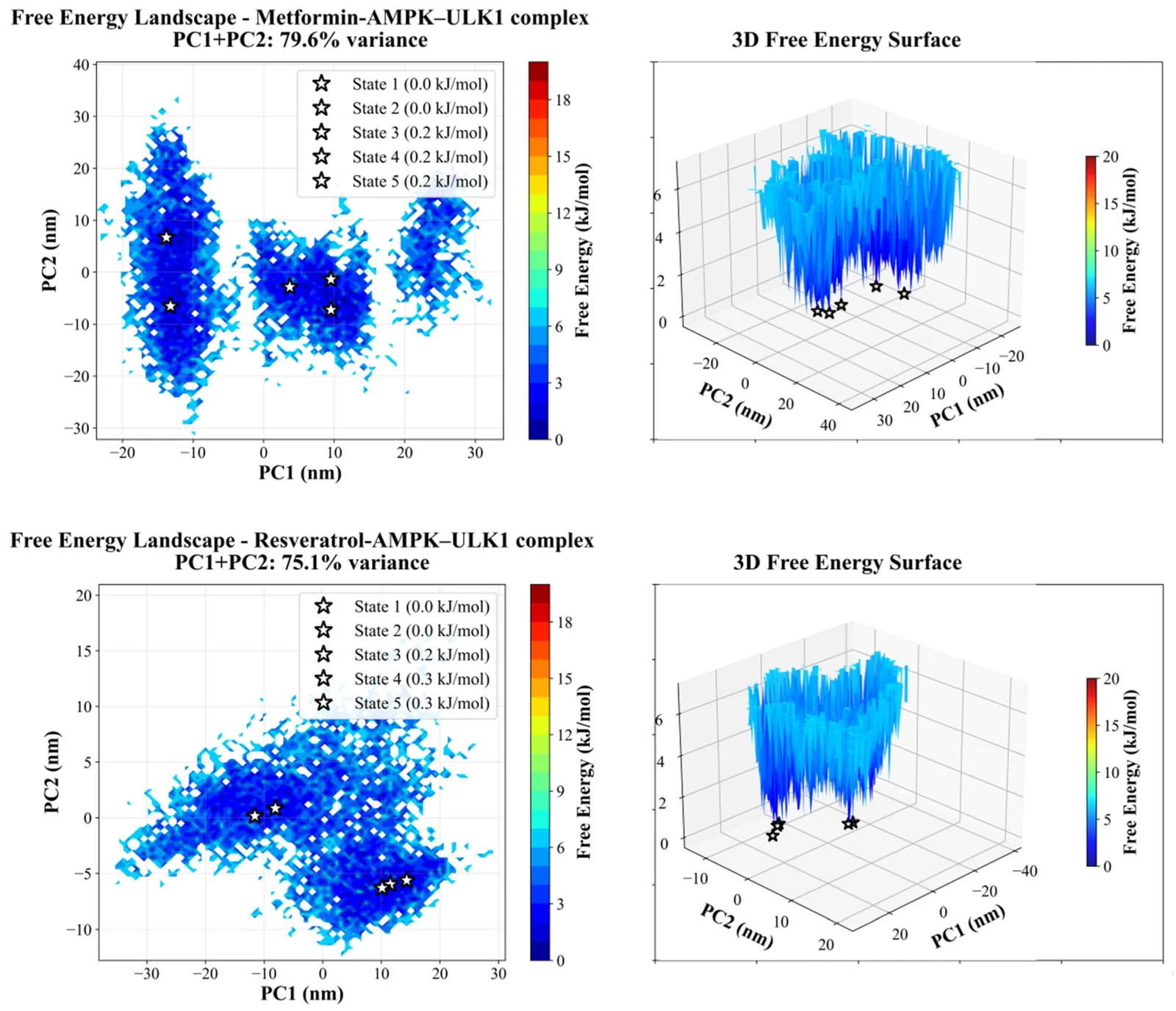
Free energy landscapes (FEL) of the AMPK–ULK1 system. The 2D contour maps (**left**) and 3D surface projections (**right**) depict the distribution of free energy as a function of the first two principal components (PC1 and PC2). The presence of multiple energy minima (denoted by stars) and varying basin depths demonstrates the thermodynamic favorability and conformational diversity of the apoprotein, curcumin, metformin, and resveratrol-bound complexes throughout the simulation.

Principal component analysis (PCA) and free energy landscape (FEL) analyses reveal that ligand binding restricts AMPK–ULK1 flexibility into dominant conformational modes, with PC1+PC2 variance higher in ligand-bound complexes (75.1–89.0%) than in the apoprotein (60.3%). Metformin (488) and resveratrol (512) complexes sample more energy minima with lower barrier heights (6.8–7.6 kJ·mol^−1^), indicating multiple accessible states and facile interconversion compared to the apoprotein and curcumin (Table 1). The FEL 2D contour and 3D surface maps (Figure 4) show deeper blue basins representing stable conformations, with curcumin exhibiting the highest variance (89.0%), indicating confinement to a well-defined conformational set; collectively, ligands stabilize specific structural basins and guide transitions to energetically favorable states. Radius of gyration (Rg) analysis (Supplementary Figure S7) demonstrates that resveratrol and curcumin maintain more compact, stable tertiary structures, whereas metformin shows slightly higher, more variable Rg, reflecting dynamic adaptation without global unfolding. Solvent-accessible surface area (SASA) analysis (Supplementary Figure S8) confirms stable profiles across all systems, with resveratrol and curcumin showing slightly lower, more consistent SASA values indicative of tighter packing, while metformin exhibits modest transient increases in solvent exposure that do not compromise structural integrity. Overall, ligand binding preserves the global AMPK–ULK1 fold while promoting compactness (resveratrol/curcumin) or adaptive flexibility (metformin).

### 3.2. PolyQ (80Q) Expression Impairs Cellular Growth and Induces Amyloid-like Aggregation in Dictyostelium discoideum

**Figure 5:**
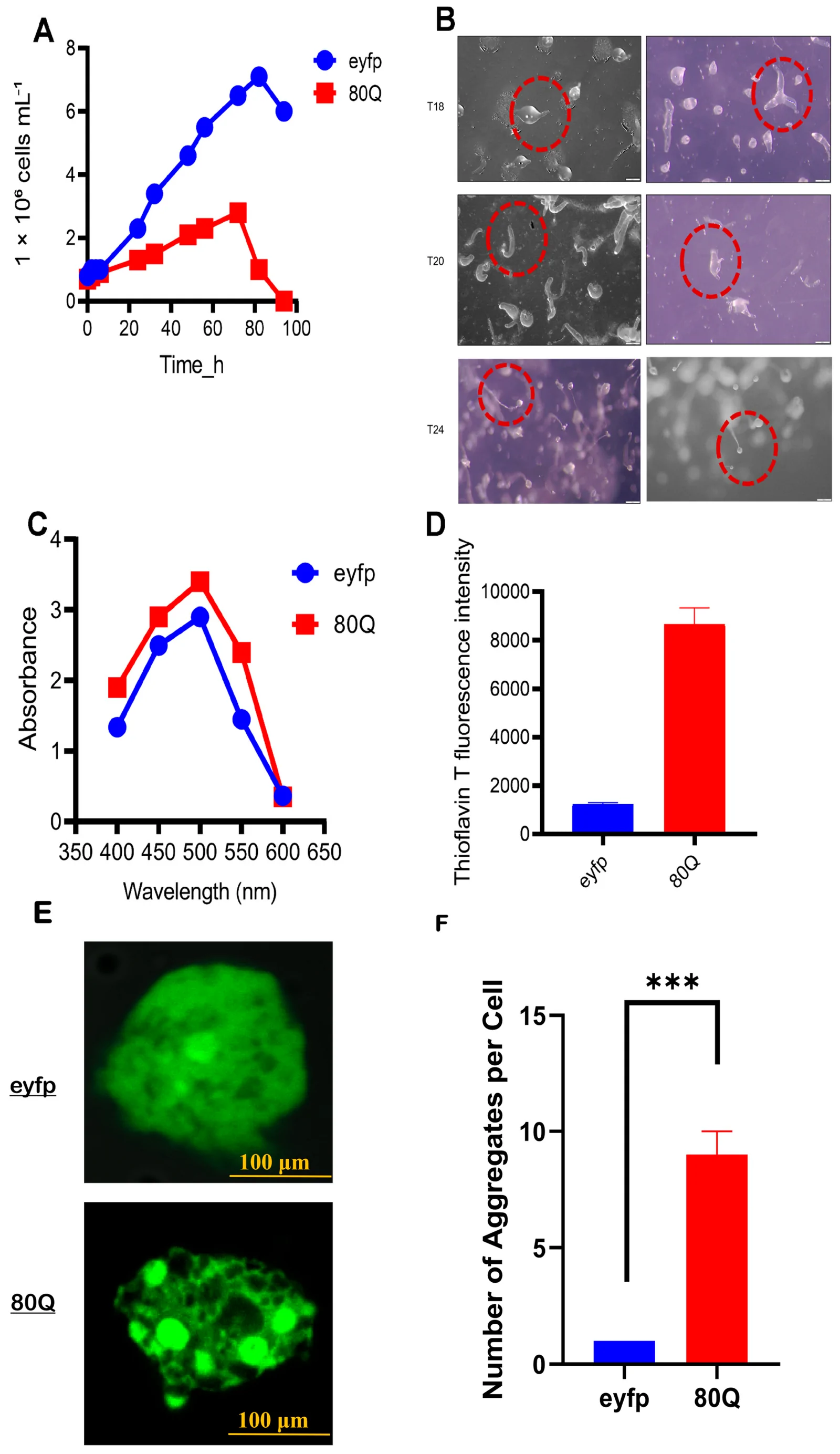
PolyQ (80Q) expression inhibits growth and induces amyloid-like aggregation in *D. discoideum*. (**A**) Growth curves of eYFP control and eYFP-80Q strains; eYFP reaches ~7 × 10^6^ cells mL^−1^ while 80Q plateaus at ~3 × 10^6^ cells mL^−1^. Calculated doubling times: ~12 h (eYFP) and ~32 h (80Q). (**B**) Aggregate quantification during development: 80Q cultures show fewer apparent aggregates and reduced mean aggregate/cell size, with aggregates producing pronounced local cytoplasmic deformation (mean ± SD; *n* = 3 replicates) (scale bar = 100 µm). (**C**) Congo Red absorbance assay (spectra): 80Q displays a shifted absorbance spectrum (400–600 nm) versus eYFP, consistent with amyloid binding. (**D**) Thioflavin-T (ThT) binding assay (spectra): 80Q cells show ~10-fold higher ThT fluorescence intensity versus eYFP (*p* < 0.001), indicating increased cross-β amyloid-like material. Excitation 440 nm, emission 480 nm. (**E**) Confocal fluorescence images: eYFP is diffuse, eYFP-80Q shows bright punctate inclusions (scale bar = 100 µm). (**F**) Quantitative confocal analysis of aggregate load: left, mean aggregates per cell; middle, mean fluorescence per aggregate; right, total aggregate fluorescence per cell (*** indicates *p* < 0.001). (The red dashed circles indicate representative *Dictyostelium discoideum* structures exhibiting characteristic morphological changes during development).

Successful generation of the 80Q-expressing strain was first confirmed by molecular cloning and stable transformation of *D. discoideum* cells with the eYFP-80Q construct. Transformed cells were selected under antibiotic pressure and maintained as stable cell lines for subsequent analyses. To determine any impact on cell proliferation due to the expression of an expanded polyglutamine repeat in *D. discoideum*, a comparison was made between cultures expressing eYFP alone and those expressing 80Q fused to eYFP in terms of growth kinetics, aggregate formation, fluorescence spectroscopy, amyloid staining, and confocal microscopy. Growth kinetic analysis showed that cultures expressing eYFP alone grew exponentially and reached approximately 7 × 10^6^ cells mL^−1^, whereas cultures expressing eYFP-80Q reached only 3 × 10^6^ cells mL^−1^, indicating that expression of the expanded polyglutamine tract impaired cellular proliferation (Figure 5A). Since the doubling time of the 80Q strain (approximately 32 h) was much greater than that of the eYFP control (approximately 12 h), expression of the expanded polyQ sequence significantly reduces the ability to divide and proliferate. Growth inhibition is consistent with the hypothesis that misfolding and accumulation of expanded polyQ proteins interferes with protein homeostasis, cell cycle regulation, and metabolic function. Microscopic and quantitative studies revealed that eYFP-80Q caused formation of punctate inclusions, whereas control cells had a diffuse pattern of eYFP (Figure 5B). The quantitative analysis of average inclusion counts and size showed that, within the context of the investigated development process, the cells expressing 80Q had fewer inclusion numbers and smaller inclusion size compared to the eYFP positive control group (Figure 5B). Despite this, 80Q inclusions did cause noticeable changes in the cellular cytoplasm organization, evidenced by irregular inclusion shapes and localized clearings around them, pointing at cytoplasm remodeling and sequestration of cell components. The spectroscopy study of cell lysate demonstrated the presence of one maximum absorption between 470 and 500 nm in case of each strain tested, which was characteristic of the eYFP chromophore (Figure 5C). The absorption was approximately 25% higher in 80Q samples compared to that in eYFP controls within the range of visible spectrum. This effect can be attributed to the environmental changes in the chromophore structure due to aggregation, scattering of light or higher extinction coefficient as a result of protein complex formation. To investigate the structural features of inclusion bodies, the cells were labeled with specific fluorescent dye Thioflavin T (ThT).

ThT fluorescence in cells with expression of 80Q was much higher than the level detected in the eYFP group (ten times more; *p* < 0.001) (Figure 5D). Since ThT fluorescence is significantly intensified by cross-β amyloid fibrils, whereas it does not fluoresce in the presence of amorphous aggregates, one may suggest that inclusion bodies of eYFP-80Q contain predominantly β-sheets. The differences were confirmed by confocal microscopy analysis (Figure 5E,F). The control cells carrying the eYFP tag showed homogenous distribution of the fluorescent protein in the cytoplasm, while the eYFP-80Q cells revealed bright, heterogeneous inclusions with punctate distribution. Quantitative analysis of the imaging data showed that there was an increased number of aggregates present in the 80Q sample, in comparison to the eYFP control group. The multi-fold increase in total aggregate fluorescence per cell (sum intensity) was seen in eYFP-80Q cells. The results from imaging are in support of growth inhibition, changes in spectroscopy, and positive Thioflavin T staining seen in the 80Q mutant strain (Figure 5). Western blotting showed that cells expressing expanded polyglutamine (80Q) had reduced total AMPK and phosphorylated AMPK (AMPK-P) compared with eYFP controls (Supplementary Figure S9A). β-actin was used for the loading control. Densitometry after β-actin normalization confirmed a significant decrease in total AMPK in 80Q cells (Supplementary Figure S9B) and a corresponding reduction in AMPK-P (Supplementary Figure S9C). These results indicate that 80Q expression lowers AMPK abundance and activation, consistent with the attenuation of AMPK signaling in this model.

### 3.3. Metformin, Curcumin, and Resveratrol Partially Rescue 80Q-Induced Growth Inhibition and Developmental Defects in Dictyostelium discoideum

For the assessment of the ability of small molecule inhibitors of cellular stress responses to reduce the toxic effects of polyQ (80Q) toxicity on *D. discoideum*, an analysis of the effect of metformin, curcumin, and resveratrol was performed using IC50 dose–response experiments based on the number of cells, and concentrations from the linear part of the sigmoidal curve (Supplementary Figure S10) were obtained at 10 µM for metformin, 45 µM for curcumin, and 100 µM for resveratrol. The viability of cells treated with these concentrations was above 50% after 48 h when compared to controls (Supplementary Figure S10).

#### 3.3.1. Drug Effects on Growth

**Figure 6:**
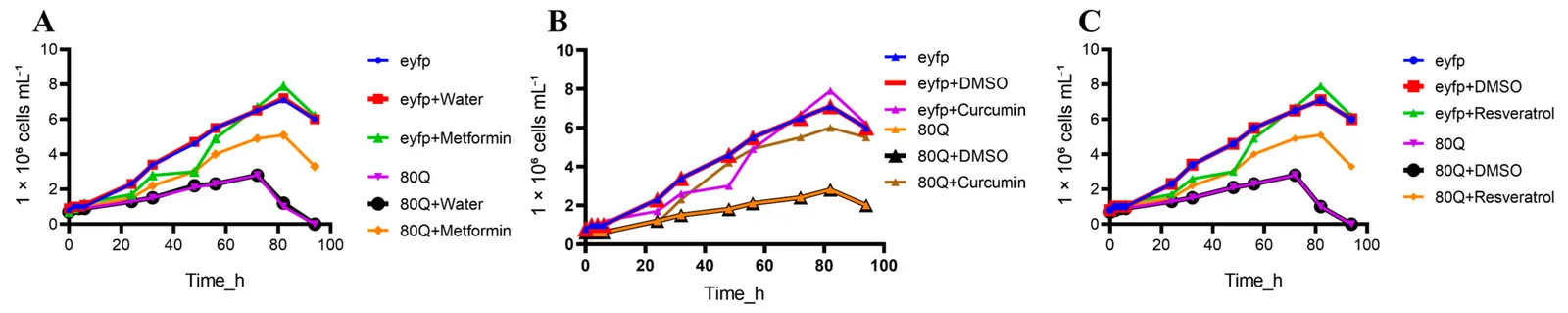

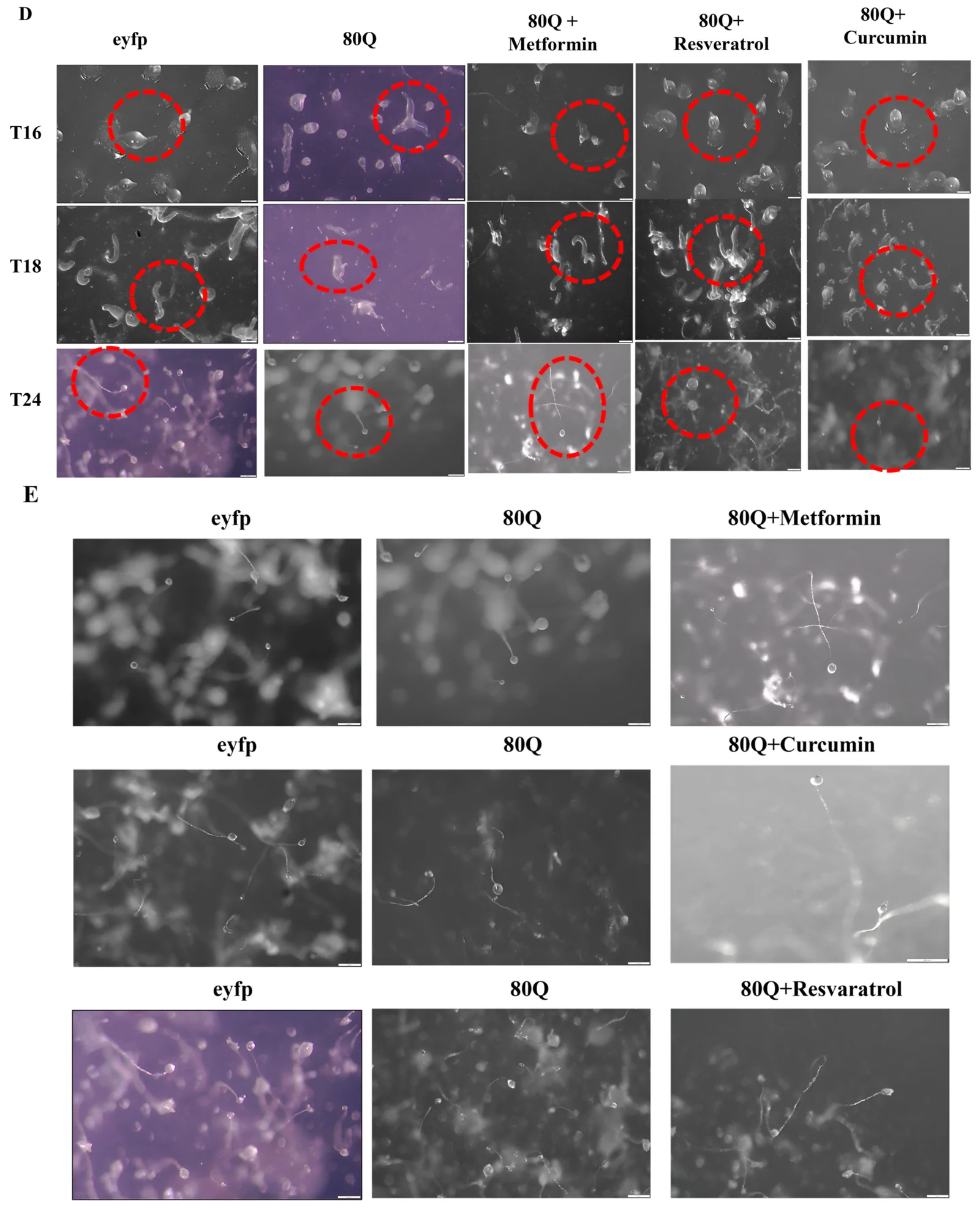

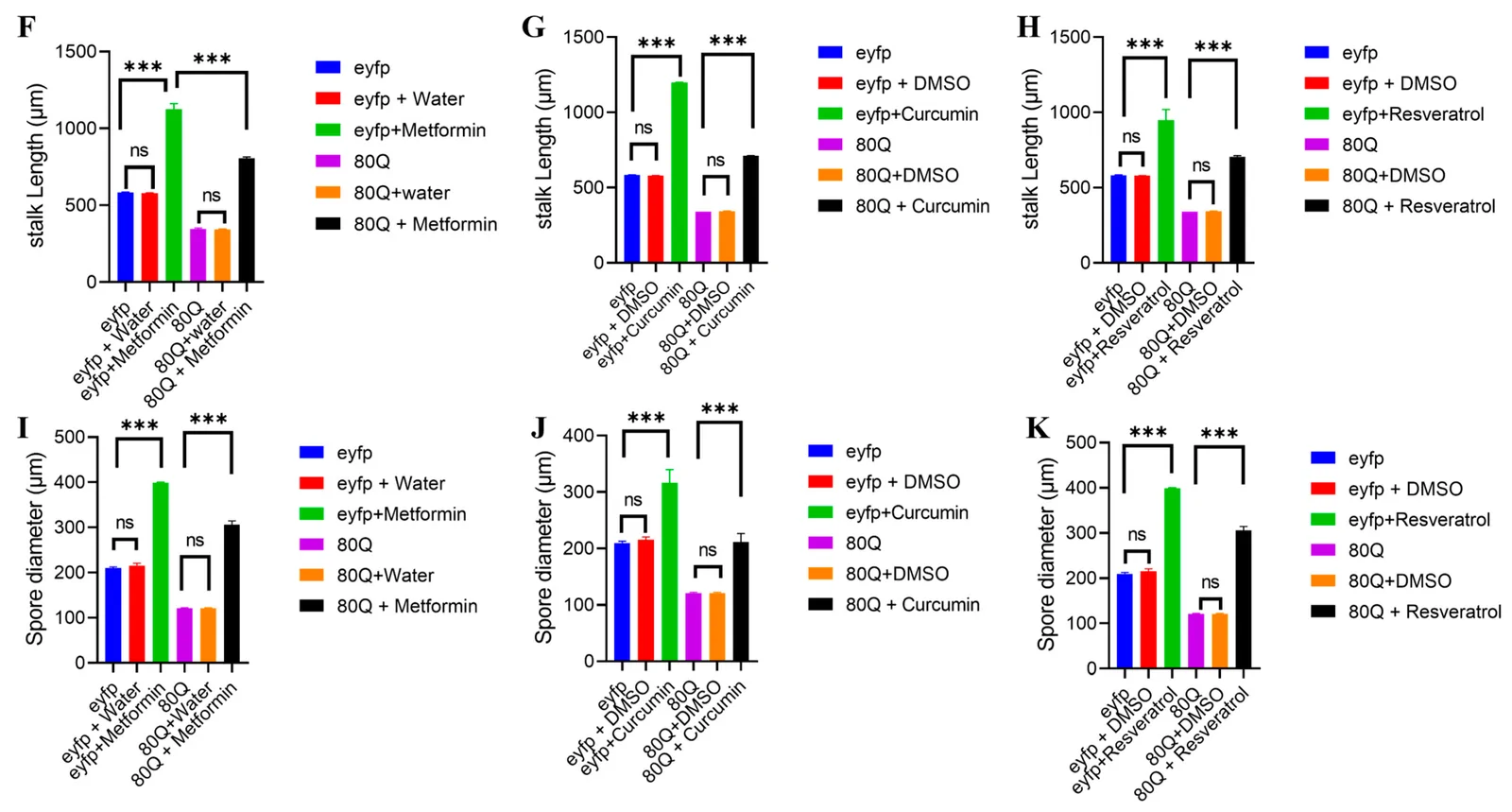
Pharmacological rescue of 80Q-induced growth and developmental defects in *D. discoideum*. (**A**–**C**) Growth curves of eYFP and eYFP-80Q cells treated with metformin, curcumin, or resveratrol. (**D**,**E**) Representative images of developmental morphology and mature fruiting bodies. (**F**–**H**) Quantification of stalk length and (**I**–**K**) spore diameter under the indicated treatment conditions. Data are presented as the mean ± SD from three independent experiments (n = 3) (scale bar = 100 µm). *** indicates *p* < 0.001; ns indicates not significant. (The red dashed circles indicate representative *Dictyostelium discoideum* cells/structures exhibiting characteristic morphological changes during development).

Growth curve analysis revealed that the growth rate was substantially decreased when cells overexpressed eYFP-80Q relative to the control cells overexpressing eYFP in all conditions tested (Figure 6A–C). Exposure to each drug resulted in a statistically significant partial rescue from the growth arrest caused by 80Q; specifically, treatment with metformin, curcumin, and resveratrol resulted in 50%, ~49%, and ~40% recovery, respectively (compared to the 80Q untreated group, *p* < 0.001). Even though no drug could fully normalize growth rates back to normal levels, the trend seen in all biological replicates suggests specific action on the polyQ-mediated growth inhibition problem. Subsequently, 80Q expression significantly increased doubling time, indicating slower growth than eyfp control cells. Metformin, curcumin, and resveratrol each reduced this elevated doubling time, showing a partial rescue of the growth defect (Supplementary Figure S11; *p* < 0.001).

#### 3.3.2. Drug Effects on Multicellular Development and Culmination

Expression of 80Q severely impaired multicellular development: 80Q strains displayed delayed aggregation, fewer and disorganized mounds and slugs, short and disordered culmination structures, and roughly a >60% reduction in formed structures per field and ~40% lower developmental efficiency at 24 h post-starvation compared with wild-type and empty-vector controls (Supplementary Figures S12 and S13; *p* < 0.001). High-resolution time-course imaging of culmination showed that eYFP controls formed tall, well-oriented fruiting bodies with distinct tips and continuous stalks, whereas 80Q culminants remained short, thick, and disorganized (Figure 6D,E).

All three compounds improved the developmental progression of 80Q cultures, producing progressively more elongated and tapered culminants with improved orientation relative to untreated 80Q (Figure 6D). Metformin produced the largest increase in the number of formed multicellular structures (≈45% restoration of structures relative to untreated 80Q; *p* < 0.001), whereas resveratrol and curcumin produced more modest but significant improvements (≈32% *p* < 0.01 and ≈28%, *p* < 0.05, respectively). These data indicate that the drugs act to restore morphogenetic programs disrupted by polyQ expression.

#### 3.3.3. Stalk Length and Spore Maturation

Drug-dependent recovery of the process of terminal differentiation was established quantitatively. The stalk lengths of 80Q fruiting bodies were significantly shorter than that of eYFP control samples (*p* < 0.001). Treatment with metformin and curcumin resulted in the 65% and 70% increase in 80Q stalk length, respectively, close to that observed in the control group; whereas resveratrol caused an approximate 50% increase in stalk length (*p* < 0.001 for the comparison of drugs vs. untreated 80Q) (Figure 6F–H). In addition, 80Q constructs displayed decreased spore size compared to those of eYFP constructs, implying poor sporulation in these strains. The spore size was enhanced significantly when drug treatments were applied to 80Q constructs: metformin approximately 45% increase, curcumin approximately 55% and resveratrol approximately 40% increase compared to the vehicle treatment of 80Q controls (*p* < 0.001 for all comparisons) (Figure 6I–K). Even though drug-induced increase in the spore size in 80Q strains.

### 3.4. Metformin, Curcumin, and Resveratrol Reduce 80Q-Dependent Amyloid Burden as Assessed by Thioflavin-T Fluorescence and Congo Red

**Figure 7:**
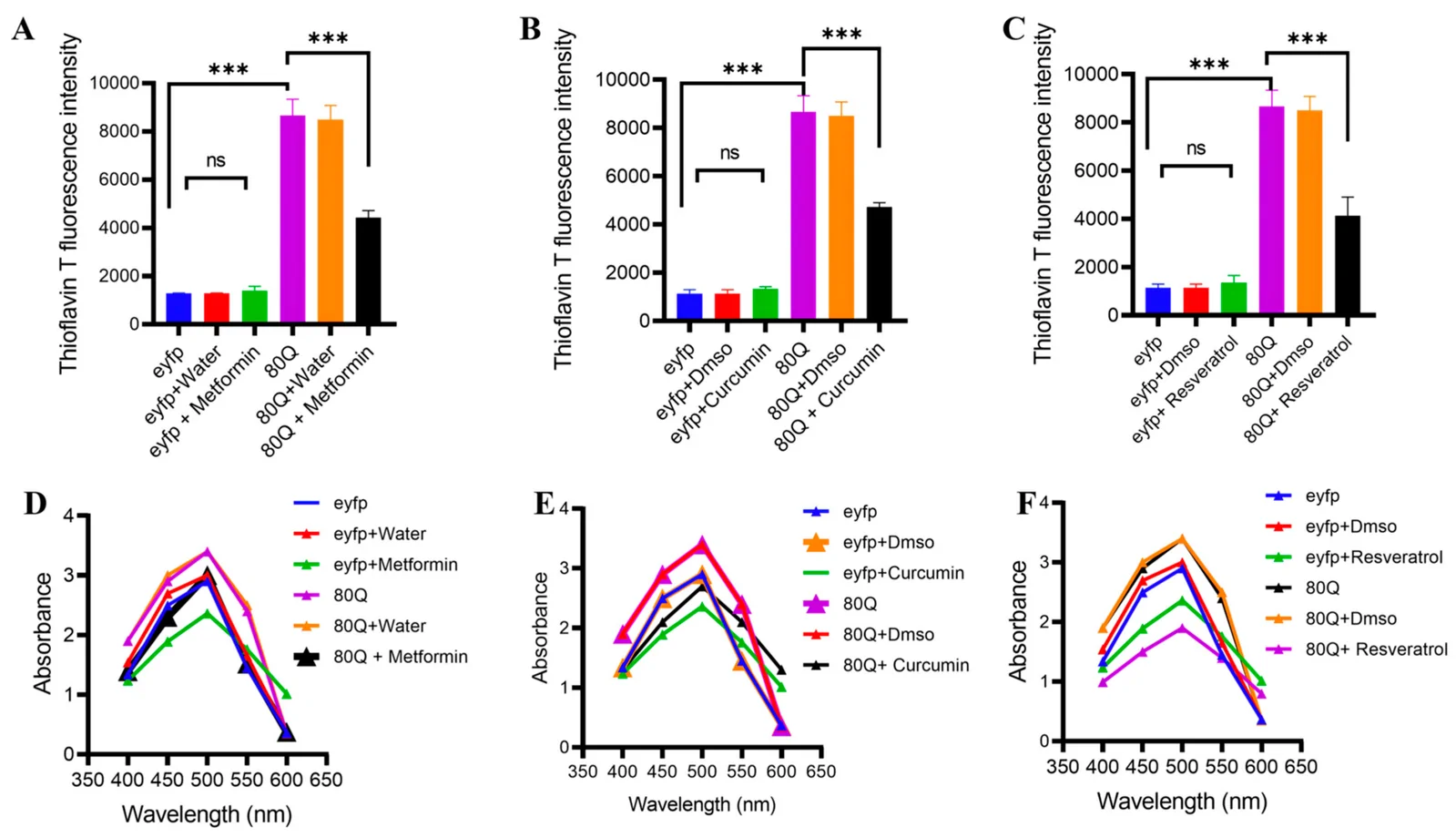
Pharmacological reduction in PolyQ amyloid burden in eYFP and eYFP-80Q cells. (**A**–**C**) Thioflavin T (ThT) fluorescence analysis of cells treated with metformin, curcumin, or resveratrol. (**D**–**F**) Representative Congo red absorbance spectra under the indicated treatment conditions. Data are presented as the mean ± SD from three independent experiments (n = 3), *** indicates *p* < 0.001; ns indicates not significant.

To determine whether the observed morphological and developmental improvements correspond to reductions in amyloid-like aggregates, we measured Thioflavin T (ThT) fluorescence and Congo Red absorbance as complementary, amyloid-sensitive readouts. In every ThT assay carried out (Figure 7A–C), both the control eYFP samples (eYFP +/− drug or vehicle) showed low fluorescence, indicating that there was no significant aggregation of β-sheet-containing protein in the absence of 80Q and indicating that drugs caused no ThT-positive aggregation in the absence of 80Q. When eYFP-80Q samples that were not treated were compared with eYFP-80Q samples treated with the various drugs, the Fluo measurements were significantly decreased in a drug-specific fashion. Metformin decreased the fluorescence by 50% versus the eYFP-80Q samples (*p* < 0.001) and curcumin decreased the fluorescence by 60% (*p* < 0.001). and resveratrol decreased ThT signal by ~40% (*p* < 0.001) (Figure 7A–C). The above results indicate that all the molecules have a capacity to lower the total cellular amyloid load created by the extended polyQ chain, whereby curcumin is the most potent among the three. An independent determination of the load through Congo Red absorbance confirmed this observation (Figure 7D–F). The samples showing the expression of 80Q displayed an expected increase in Congo Red absorbance within the range of 450–550 nm in comparison with the eYFP controls due to increased interaction of Congo red with amyloids. In quantitative terms, the absorbance of Congo Red at 450–550 nm was nearly doubled for 80Q in comparison to that for eYFP. Metformin shifted the 80Q spectrum towards the one of eYFP, meaning there was significant, but partial, elimination of the aggregates detected by Congo Red. The curcumin-induced change in the 80Q spectrum was the most dramatic; it was almost identical to the eYFP one, which indicates that curcumin efficiently prevents the aggregation of Congo Red-positive amyloids. The resveratrol had an intermediate effect, where the spectrum of 80Q plus resveratrol was situated in between the spectrum of untreated 80Q and eYFP. In the wavelength range of 450 to 550 nm, there was a 20–40% decrease in Congo Red absorbance for all three samples compared with the untreated 80Q sample.

### 3.5. PolyQ Expression Induces Autophagy-Related Gene Expression That Is Normalized by Metformin, Curcumin, and Resveratrol

**Figure 8:**
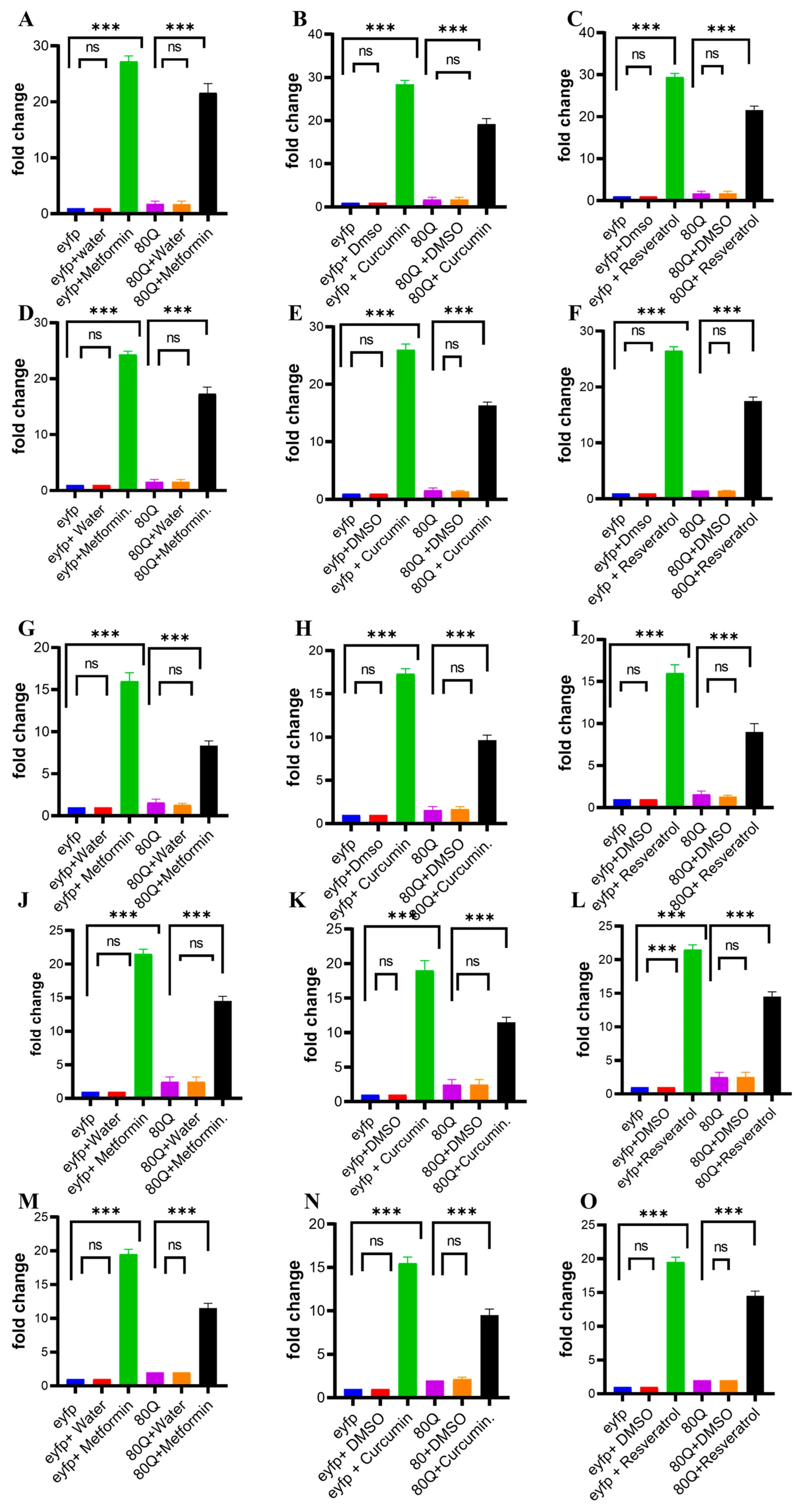
Expression of autophagy-related genes in eYFP and eYFP-80Q cells following treatment with metformin, curcumin, or resveratrol. (**A**–**F**) Relative expression of *ATG1* and *ATG5*. (**G**–**L**) Relative expression of *ATG8* and *ATG9*. (**M**–**O**) Relative expression of *ATG16* in eYFP and 80Q cells following treatment with metformin, curcumin, or resveratrol. Data are presented as mean ± SD (n = 3). Statistical analysis was performed using one-way ANOVA followed by Tukey’s multiple comparison test. *** *p* < 0.001.

Treatment with metformin, curcumin, or resveratrol markedly suppressed the induction of these autophagy gene expression caused by 80Q mutation, while not impacting the expression level under normal conditions (eYFP control) (Figure 8A–O). In particular, *ATG1* and *ATG5* (Figure 8A–F) metformin, curcumin, and resveratrol were able to inhibit *ATG1* and *ATG5* expression in mutant cells compared with their respective controls (*p* < 0.001); however, no inhibition was seen in eYFP cells with respect to these proximal autophagy regulators (ns). The normalized expression level for these proximal autophagy regulators implies that these drugs reduce the autophagy signal caused by polyQ-induced stress; probably through regulating upstream nutrient/energy sensors (e.g., *AMPK-mTOR* pathway). *ATG8* and *ATG9* (Figure 8G–L) A significant increase in the expression levels of *ATG8* and *ATG9* was noted in the 80Q treatment group compared with eYFP (*p* < 0.001). This increase was inhibited by metformin, curcumin, and resveratrol in the 80Q treatment group towards normal levels (*p* < 0.001 vs. 80Q without treatment; no significant difference from eYFP), suggesting that signaling of phagophore extension and the necessity of delivering membranes to form the autophagosome were being attenuated. *ATG16* (Figure 8M–O) An induction of *ATG16* expression was observed with the 80Q treatment compared with the eYFP control (*p* < 0.001). Significant inhibition of *ATG16* expression was seen in the 80Q treatment group after treatment with all three compounds (*p* < 0.001) without affecting baseline levels of eYFP expression.

### 3.6. Metformin, Curcumin, and Resveratrol Modulate mTORC1 Signaling by Suppressing Raptor and Enhancing TSC2 Expression in 80Q Cells

**Figure 9:**
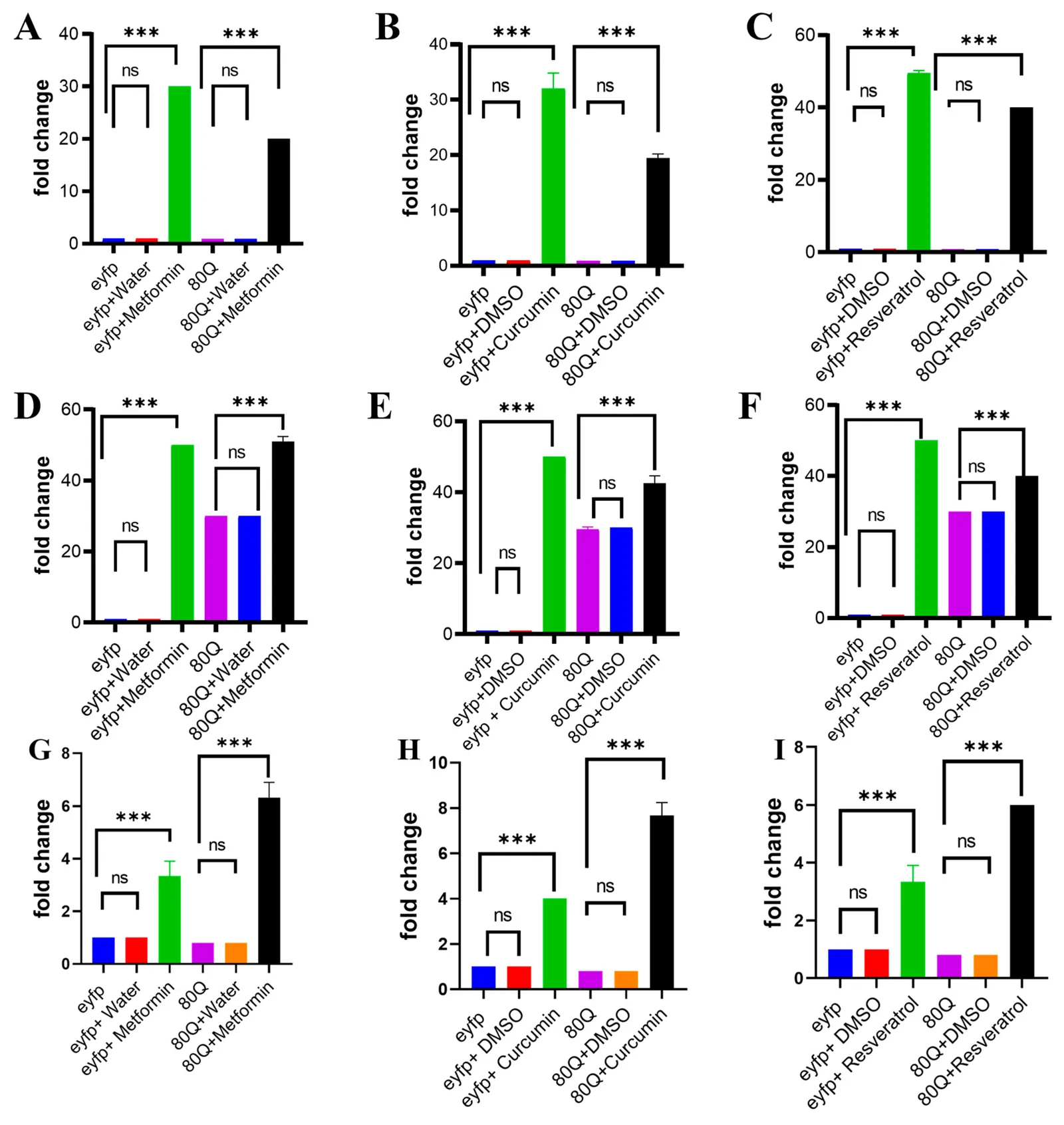
Effects of metformin, curcumin, and resveratrol on mTOR pathway-associated genes in eYFP and eYFP-80Q cells. (**A**–**C**) Relative raptor mRNA expression and (**D**–**F**) relative TSC2 mRNA expression determined by RT-qPCR. (**G**–**I**) Relative AMPK mRNA expression levels under the indicated treatment conditions. Data are presented as the mean ± SD from three independent experiments (n = 3), *** indicates *p* < 0.001; ns indicates not significant.

In order to explore how changes in polyQ sequences affect the regulatory pathway for nutrient sensing and growth control, the relative mRNA levels of important regulators involved in the process (*Raptor* and *TSC2*) were measured in both eYFP control and eYFP-80Q strains in the presence and absence of metformin (10 μM), curcumin (45 μM), or resveratrol (100 μM). In the case of biological repeats, increased Raptor levels were seen as a direct result of 80Q expression compared to eYFP strains (*p* < 0.001) (Figure 9A–C). Consistent with the observed transcriptional changes, treatment with metformin, curcumin, and resveratrol resulted in a significant reduction in raptor mRNA expression compared with corresponding untreated 80Q cells (*p* < 0.001), with decreases of approximately 35–50%. In contrast, these treatments did not significantly affect Raptor transcript levels in eYFP control cells (ns) (Figure 9A–C). These findings suggest that the compounds selectively modulate Raptor gene expression in cells expressing 80Q mutant Huntingtin rather than exerting nonspecific effects on basal transcription. Previous studies have reported that metformin, curcumin, and resveratrol are associated with modulation of AMPK signaling and mTOR-related pathways. Therefore, the observed reduction in Raptor transcript levels is consistent with altered regulation of mTORC1-associated signaling in 80Q-expressing cells. Additionally, increased expression of TSC2, a negative regulator of mTORC1, was observed in response to 80Q expression (*p* < 0.001) (Figure 9D–F). Notably, metformin, curcumin, and resveratrol each further and significantly increased *TSC2* expression in 80Q cells beyond the 80Q + vehicle baseline (*p* < 0.001). Drug-dependent enhancement of TSC2 by ~25–40% (relative to 80Q + vehicle) is consistent with amplification of the cell’s endogenous brake on *mTORC1* signaling, thereby shifting the equilibrium toward *mTORC1* suppression and promoting pro-autophagic, stress-adaptive outcomes. (Figure 9G–I) AMPK mRNA expression analysis by RT-qPCR demonstrated a significant downregulation of AMPK in the disease model compared with the control group. Treatment with metformin and the test compound markedly increased AMPK expression, restoring transcript levels toward those observed in the control group. These findings indicate that the therapeutic intervention effectively activates the AMPK signaling pathway and may contribute to the amelioration of disease-associated cellular dysfunction. Data are presented as the mean ± SD.

### 3.7. Microscopic Analysis of Aggregate Formation

**Figure 10:**
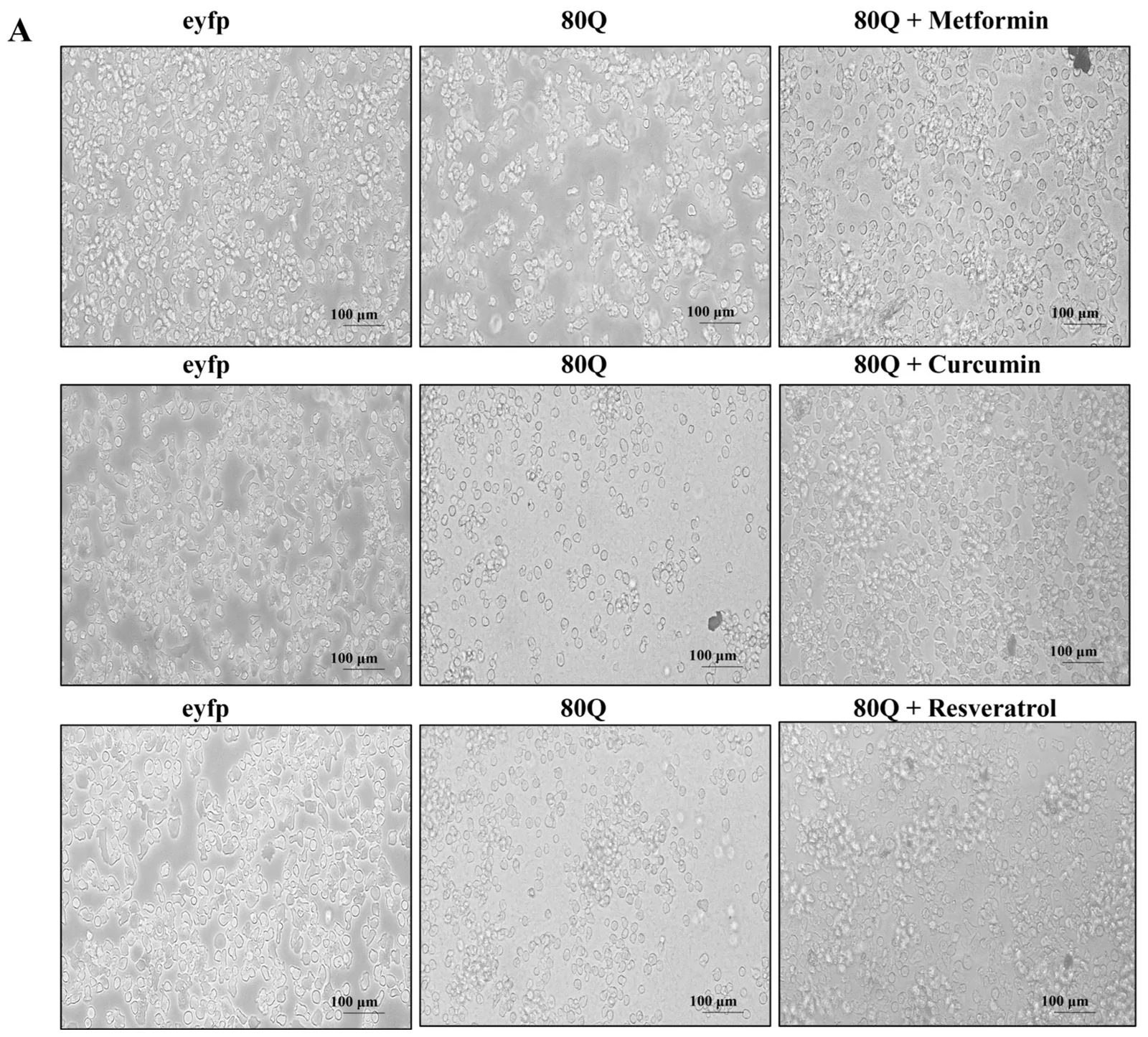

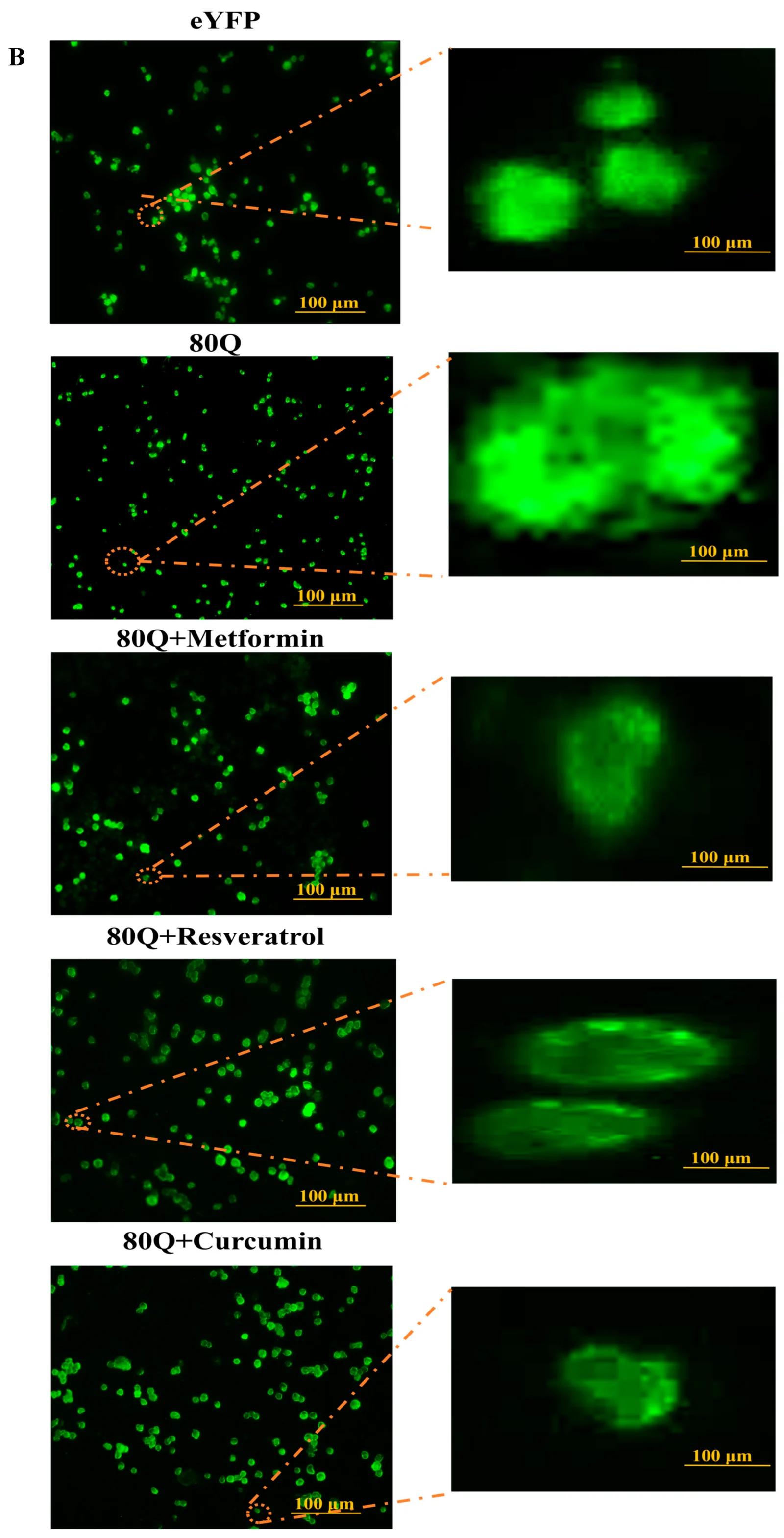

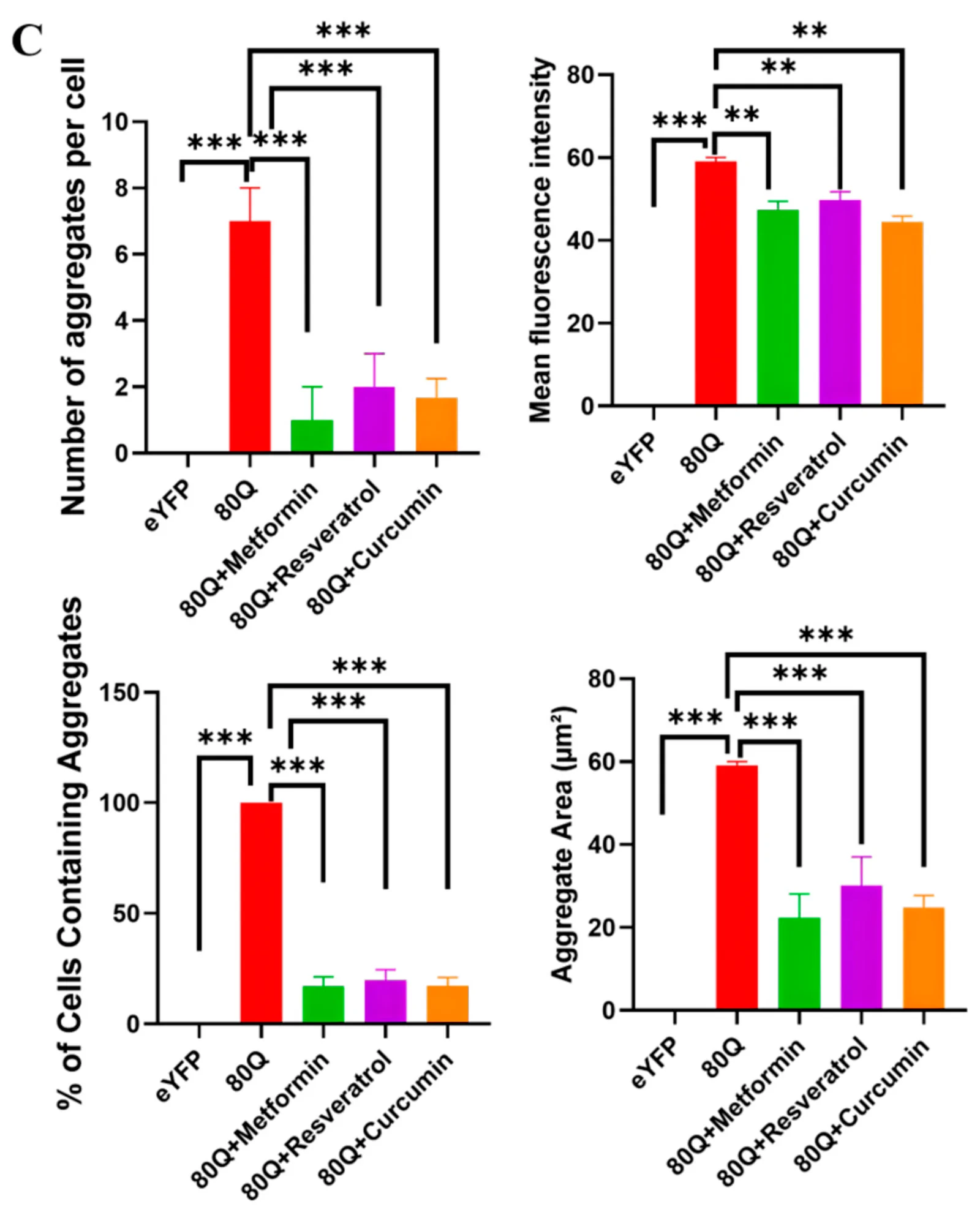
(**A**) Brightfield microscopic analysis of *D.discoideum* morphology under polyQ stress and drug treatment conditions. eYFP-expressing control cells exhibited normal cellular morphology and healthy cell density, whereas 80Q-expressing cells showed altered morphology, reduced cellular density, and signs of cellular stress. Treatment with metformin, curcumin, and resveratrol partially restored normal cellular appearance and improved overall cell distribution compared with untreated 80Q cells. Images were obtained under identical imaging conditions. Scale bar = 100 µm. (**B**) Fluorescence microscopy of intracellular aggregates. Cells under control, disease, and treatment conditions were analyzed for green fluorescent protein aggregates (scale bar = 100 µm). (**C**) The disease group showed increased aggregate number and size, whereas compound-treated groups exhibited significantly reduced aggregate burden and aggregate-positive cells, consistent with suppression of pathological protein aggregation ** *p* < 0.01, *** *p* < 0.001.

Brightfield microscopy was used to assess the impact of polyQ expression on cell growth in *D. discoideum*. EYFP control cells showed robust growth and a homogeneous cell population, whereas 80Q-expressing cells exhibited reduced cell density, indicating impaired cell proliferation and/or survival under polyQ stress. Drug-treated 80Q cells displayed improved cell growth compared to untreated 80Q cells. Among the tested compounds, metformin, curcumin, and resveratrol partially rescued the growth defect in 80Q cells, suggesting that metabolic modulators can mitigate polyQ-induced growth impairment in this disease model (Figure 10A). *D. discoideum* cells expressing eYFP (control), 80Q, or 80Q treated with metformin, resveratrol, or curcumin were imaged by brightfield microscopy, and cell number per field was manually counted. Bars represent mean cell counts per image field ± SD from three independent fields per condition. Expression of 80Q reduced cell number compared with eYFP, whereas metformin, resveratrol, and curcumin restored or increased cell counts relative to control, indicating that these compounds alleviate polyQ-induced growth impairment (Supplementary Figure S14). Fluorescence microscopy revealed a robust increase in intracellular green fluorescent aggregates in the disease/model group compared with control cells, as evidenced by more numerous, larger puncta and denser inclusions at higher magnification. Treatment with the test compounds markedly reduced aggregate burden, with one compound producing the greatest decrease in puncta number and size, indicating improved proteostasis (Figure 10B). Quantitative image analysis confirmed these observations, showing significantly elevated aggregate counts, fluorescence intensity, and percentage of aggregate-positive cells in the disease group relative to controls, all of which were significantly reduced by treatment toward control levels. These data indicate that the tested compounds attenuate pathological protein aggregation and may confer protection against aggregation-associated cellular stress (Figure 10C).

### 3.8. PPI Network Construction

The STRING analysis of proteins involved in the AMPK–autophagy pathway demonstrates that there is a strong functional connection between the core components of this pathway such as ATG1, TOR, RAPTOR, and PRKAG. The interactions between the proteins indicate the possibility of regulation by AMPK of autophagy, which is an important process for removing aggregated proteins (Supplementary Figure S15).

## 4. Discussion

In this study, we characterized the cellular and developmental consequences of expressing an expanded polyglutamine tract (80Q) fused to eYFP in *D. discoideum* and tested whether three small molecules—metformin, curcumin, and resveratrol—could mitigate the resulting phenotypes [50,51,52]. Expression of eYFP-80Q markedly impaired proliferation and multicellular development, promoted the formation of intracellular inclusions with amyloid-like properties, as shown by Thioflavin T and Congo red positivity and altered eYFP absorbance, and induced a broad transcriptional stress response that included robust upregulation of core autophagy genes and mTORC1-associated regulators [53,54,55,56]. Metformin, curcumin, and resveratrol produced consistent and reproducible partial rescue across biochemical, molecular, and morphological endpoints: they reduced amyloid load, lowered stress-induced ATG gene expression, rebalanced mTORC1-related signaling by decreasing Raptor and increasing TSC2, and improved growth, aggregate formation, stalk elongation, and spore maturation [56,57,58,59,60,61,62,63]. Collectively, these results support a model in which 80Q aggregation imposes proteostatic and energetic stress that activates autophagy-related transcriptional programs and maladaptive mTORC1 signaling; pharmacological interventions that modulate energy sensing and proteostasis partially restore homeostasis and developmental competence [64,65,66,67].

The observed growth inhibition and prolonged doubling time of eYFP-80Q cells are consistent with a cellular burden imposed by misfolded polyQ species and their assemblies. PolyQ expansions are known to impair proteostasis by sequestering chaperones, overloading degradation systems, and perturbing organelle function [53,54,68,69,70,71]. Our confocal imaging, ThT staining, and Congo red absorbance together indicate that the 80Q inclusions in *D. discoideum* adopt β-sheet-rich, amyloid-like structures rather than amorphous deposits [72,73,74,75]. Spectral perturbations of the eYFP chromophore in aggregated samples further support the presence of tightly packed higher-order assemblies that alter the fluorophore’s environment and light scattering. In the developmental context, these molecular lesions manifest as delayed aggregation, defective slug and culmination formation, shortened stalks, and smaller spores. Such morphogenetic defects likely arise from multiple convergent mechanisms, including impaired cell–cell signaling or chemotaxis during aggregation, energy deficits that compromise differentiation programs, dysregulated autophagy or cell death leading to cell loss or dysfunction, and sequestration of proteins required for terminal morphogenesis [76,77,78].

All three tested compounds produced reproducible partial rescues across endpoints, but with distinct magnitudes and biochemical signatures [55,56,57,58,59]. Curcumin yielded the strongest reduction in amyloid measures and the most complete normalization of Congo red spectra, metformin produced broad improvements in growth and structure formation, and resveratrol provided moderate benefits. Mechanistically, the concordant effects on ATG transcript levels, Raptor downregulation, and TSC2 upregulation indicate that these drugs act, at least in part, by modulating the AMPK–mTORC1 axis and downstream autophagy [60,61,62,63,67]. Metformin is a recognized AMPK activator and indirect mTOR inhibitor in many systems. In our data, metformin reduced Raptor abundance and increased TSC2 in 80Q cells, consistent with enhanced negative regulation of mTORC1 and promotion of autophagic activity. Curcumin and resveratrol have both been reported to modulate AMPK, SIRT1, and PI3K–AKT signaling and to exert anti-aggregation and antioxidant effects [56,58,66,79,80,81,82,83,84]. Curcumin’s potent biochemical normalization of Congo red and ThT signals may reflect a combination of direct anti-amyloid activity and enhancement of aggregate clearance pathways. Resveratrol’s moderate effects are compatible with its pleiotropic modulation of sirtuins, AMPK, and antioxidant defenses, which may partially alleviate proteostatic stress but at lower potency under the tested conditions.

The coordinated partial downshift in multiple ATG *genes (ATG1, ATG5, ATG8, ATG9, ATG16)* indicates that drug treatment restores autophagy from a hyperactivated or dysregulated state to a more homeostatic level rather than simply blocking autophagy. This distinction is crucial because excessive or maladaptive autophagy can be deleterious, whereas appropriate autophagic activity is required for aggregate clearance and cellular survival. The combination of reduced amyloid load with rebalanced autophagy gene expression suggests that these compounds may both enhance aggregate removal and dampen the chronic stress signal that drives autophagy overdrive [59,60,61,62,66]. In this context, resveratrol has been reported to promote neuroprotection and autophagy-related clearance in models of protein aggregation, including mutant huntingtin systems, supporting the plausibility of the response observed here. Likewise, curcumin has previously been shown to modulate cell death and protect in Huntington’s disease models, as well as promote autophagic flux and aggregate clearance in neuronal cells.

The core AMPK–TSC2–mTORC1–ULK1 network and its extended connections to PI3K/AKT, FOXO, SIRT1, and canonical autophagy effectors align with our experimental observations [60,61,62,63]. This systems perspective explains why drugs with diverse primary activities can produce overlapping phenotypic outcomes: they converge on conserved nodes that coordinate energy status, proteostasis, and autophagic clearance [62,63,64,65,69]. The present findings therefore add to the growing evidence that AMPK-centered modulation may represent a general strategy to counter protein aggregation toxicity across neurodegenerative contexts [59,60,61,62,78,79,80,81,82,83]. These findings have implications for disease modeling and therapeutic discovery. *D. discoideum* provides a tractable, multicellular platform for studying aggregate proteotoxicity and the developmental consequences of polyQ expansion [74,75,76,77,78]. Our data demonstrate that fundamental aspects of polyQ aggregation—amyloid formation, proteostasis stress, autophagy induction, and downstream morphological defects—are recapitulated in this organism and that conserved pharmacological modulators can partially ameliorate these perturbations [72,73,74,75,76,77,78]. Thus, *D. discoideum* represents a useful initial platform for screening and mechanistic dissection of candidate anti-aggregation compounds, particularly those targeting energy sensing and autophagy pathways [72,73,74,75,76,77,78].

Brightfield and fluorescence microscopy in *D. discoideum* show that 80Q expression reduces cell density and increases intracellular amyloid-like aggregates relative to eYFP controls, indicating impaired proliferation/survival and proteostatic stress. Treatment with metformin, curcumin, or resveratrol partially restored cell counts and markedly reduced aggregate number, size, and fluorescence intensity, suggesting that metabolic modulators ameliorate polyQ-induced growth and aggregation phenotypes. Causality between aggregate clearance and phenotypic rescue remains unproven, and *D. discoideum* lacks neuron-specific features, necessitating validation in mammalian neuronal models [74,75,76,77,78].

**Figure 11:**
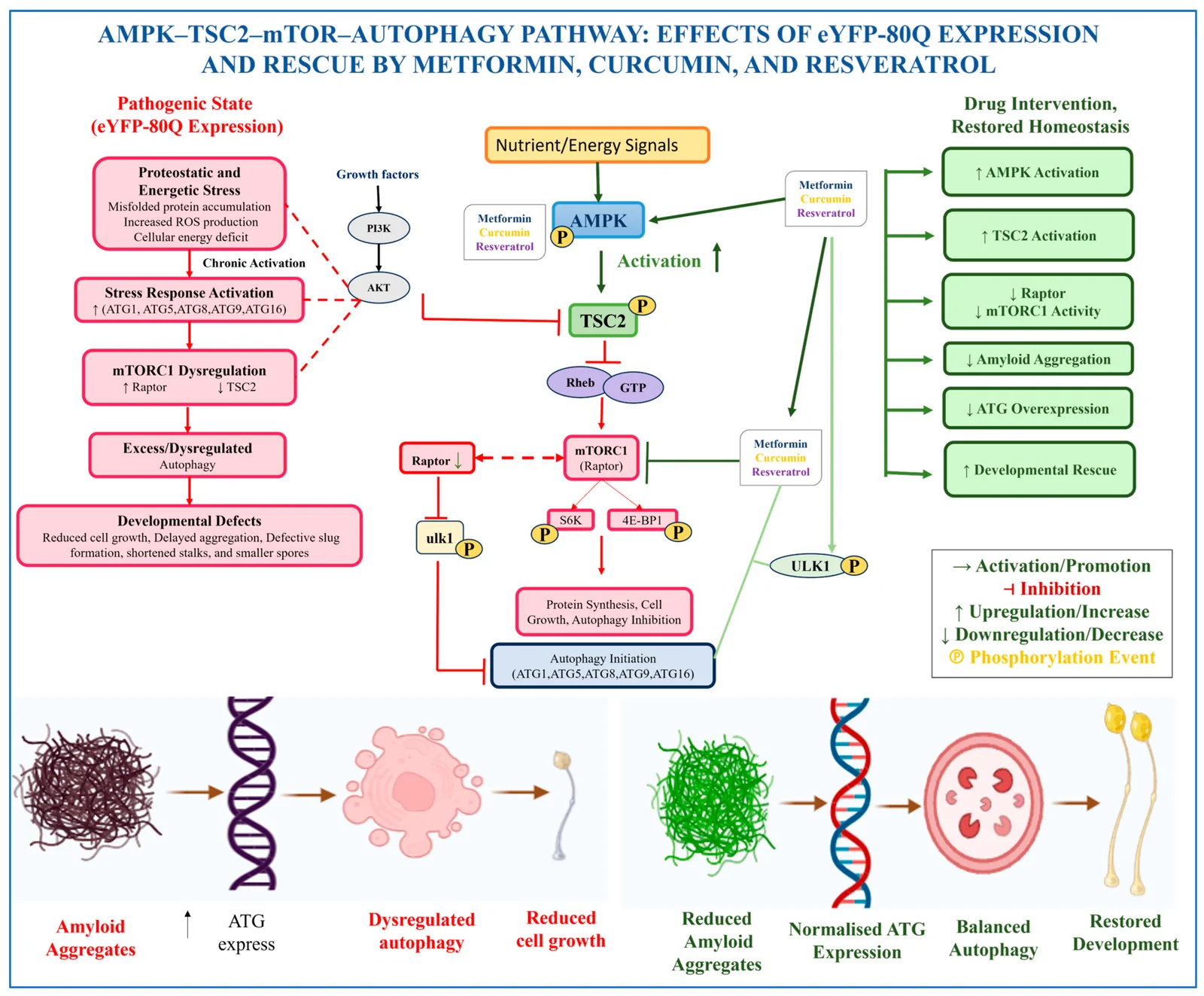
Schematic of the AMPK–TSC2–mTOR–autophagy pathway showing how eYFP-80Q induces proteostatic stress, mTORC1 dysregulation, amyloid aggregation, and developmental defects in *D. discoideum*. Metformin, curcumin, and resveratrol activate AMPK/TSC2, inhibit mTORC1 signaling, and restore balanced autophagy and normal development.

Future work should address these limitations and further probe the underlying mechanisms. Structural confirmation of fibrillar assemblies by transmission electron microscopy or cryo-EM, together with biochemical seeding assays, would strengthen the amyloid-like aggregate interpretation. Autophagic flux should be assessed using LC3 lipidation assays, p62/SQSTM1 turnover, and lysosomal inhibition to determine whether drug treatments genuinely enhance aggregate clearance. Direct signaling readouts, including phosphorylation of mTORC1 targets, AMPK-associated signaling components, and ULK1, would help clarify how these compounds influence pathway activity [60,61,62,63]. Expanded dose- and time-dependent studies, genetic perturbation of key regulators such as ATG genes, TSC2, or raptor, and validation in mammalian neuronal models would further define causality, therapeutic windows, and translational relevance [60,61,62,63,72,73,74,75,76,77,78]. Overall, the integrated phenotypic, biochemical, molecular, and systems-level analyses demonstrate that expanded polyQ expression in *D. discoideum* induces amyloid-like aggregates, proteostatic stress, autophagy-related responses, and developmental defects [53,54,55,72,73,74,75,76,77,78]. Metformin, curcumin, and resveratrol partially ameliorate these phenotypes by reducing aggregate burden and modulating autophagy- and mTOR-related pathways [57,58,59,60,61,62,63,64,65]. Collectively, these findings are consistent with the involvement of AMPK-associated signaling and autophagy-related mechanisms in regulating polyQ toxicity and support further mechanistic and translational studies focused on energy-sensing and proteostasis pathways [62,63,64,65,69]. Figure 11 summarizes the AMPK–TSC2–mTOR–autophagy pathway, showing that eYFP-80Q induces proteostatic stress and mTORC1 dysregulation leading to aggregation and developmental defects, whereas metformin, curcumin, and resveratrol restore autophagy balance via AMPK activation.

## 5. Conclusions

The current research aims to investigate the ability of metformin, resveratrol, and curcumin in reversing polyQ-induced cytotoxicity using a synthetic *Dictyostelium discoideum* cell line. Based on the results of this experiment, it has been confirmed that these metabolic regulators help in the degradation of 80Q amyloid-like aggregates via AMPK-mTOR signaling pathway-dependent restoration of autophagic flux. It should be noted that molecular docking analysis supports the existence of a stable binding of these compounds with AMPK. Through demonstrating that drug therapy could ameliorate the growth and development defects caused by 80Q expression, it becomes clear that the importance of proteostasis is proven, and treatment of polyglutamine disorders using AMPK pathway may provide a promising solution. Thus, *D. discoideum* turns out to be a cost-efficient platform suitable for the investigation of neuroprotective drugs.

## Figures and Tables

**Table 1:** Principal component analysis (PCA)-derived conformational landscape parameters of the AMPK–ULK1 complex apoprotein and ligand-bound complexes, showing cumulative PC1+PC2 variance, number of energy minima, and free energy barrier heights obtained from molecular dynamics simulations.

Sr.	Complex	PC1+PC2 Variance (%)	Energy Minima (n)	Barrier Height (kJ/mol)
1	AMPK–ULK1 apoprotein	60.3	371	7.6
2	Curcumin–AMPK–ULK1 complex	89.0	307	7.6
3	Metformin–AMPK–ULK1 complex	79.6	488	6.8

## Data Availability

The raw data supporting the conclusions of this article will be made available by the authors on request.

## Supplementary Materials

The following supporting information can be downloaded at: https://www.mdpi.com/article/10.3390/biomedicines14091931/s1.

## Abbreviations

The following abbreviations are used in this manuscript:

polyQ	Polyglutamine
80Q	80-glutamine repeat (engineered polyglutamine tract)
AMPK	AMP-activated protein kinase
mTOR	Mechanistic target of rapamycin
mTORC1	mTOR complex 1
TSC1/TSC2	Tuberous sclerosis complex 1/2
RPTOR (Raptor)	Regulatory-associated protein of mTOR
RHEB	Ras homolog enriched in brain
ULK1	Unc-51-like autophagy activating kinase 1
ATG1	Autophagy-related gene 1
ATG5	Autophagy-related gene 5
ATG7	Autophagy-related gene 7
ATG8	Autophagy-related gene 8
ATG9	Autophagy-related gene 9
ATG13	Autophagy-related gene 13
ATG16	Autophagy-related gene 16
BECN1	Beclin-1
MAP1LC3B	Microtubule-associated protein 1 light chain 3 beta
LC3	Light chain 3
SQSTM1	Sequestosome-1 (p62)
HTT	Huntingtin
AKT1	AKT serine/threonine kinase 1
PIK3CA	Phosphatidylinositol-4,5-bisphosphate 3-kinase catalytic subunit alpha
FOXO3	Forkhead box O3
SIRT1	Sirtuin 1
PRKAA1	Protein kinase AMP-activated catalytic subunit alpha 1
PRKAA2	Protein kinase AMP-activated catalytic subunit alpha 2
PRKAB1	Protein kinase AMP-activated non-catalytic subunit beta 1
PRKAG1	Protein kinase AMP-activated non-catalytic subunit gamma 1
RB1CC1	RB1-inducible coiled-coil 1
STK11	Serine/threonine kinase 11 (LKB1)
eYFP	Enhanced yellow fluorescent protein
GFP	Green fluorescent protein
YFP	Yellow fluorescent protein
CAG	Cytosine–adenine–guanine (trinucleotide repeat)
RT-PCR	Reverse transcription polymerase chain reaction
ThT	Thioflavin T
PI3K	Phosphatidylinositol 3-kinase
D. discoideum	Dictyostelium discoideum
Ax2	Axenic strain 2 (of Dictyostelium discoideum)
WT	Wild type
MD	Molecular dynamics
RMSD	Root mean square deviation
RMSF	Root mean square fluctuation
MM-GBSA	Molecular mechanics—Generalized Born surface area
MM-PBSA	Molecular mechanics—Poisson–Boltzmann surface area
PCA	Principal component analysis
FEL	Free energy landscape
PC1	Principal component 1
PC2	Principal component 2
SASA	Solvent accessible surface area
SP	Standard precision (docking mode)
PDB	Protein Data Bank
RCSB	Research Collaboratory for Structural Bioinformatics
3D	Three-dimensional
2D	Two-dimensional
PME	Particle mesh Ewald
NVT	Constant number, volume, and temperature ensemble
NPT	Constant number, pressure, and temperature ensemble
LINCS	Linear constraint solver algorithm
TIP3P	Transferable intermolecular potential with 3 points (water model)
ΔG	Gibbs free energy change
ΔEvdW	Van der Waals energy contribution
ΔEElec	Electrostatic energy contribution
ΔEPolar	Polar solvation energy contribution
ΔENonpolar	Nonpolar solvation energy contribution
Rg	Radius of gyration
SD	Standard deviation
SEM	Standard error of the mean
KEGG	Kyoto Encyclopedia of Genes and Genomes
GO	Gene Ontology
UMAP	Uniform manifold approximation and projection
LTS	Long-term support (Ubuntu version type)
IC_50_	Half-maximal inhibitory concentration
UV-VIS	Ultraviolet-visible spectrophotometry
DMSO	Dimethyl sulfoxide
mM	Millimolar
µM	Micromolar
rpm	Revolutions per minute
nm	Nanometer
Å	Angstrom
ns	Nanosecond
ps	Picosecond
