# Supplementary material for: Drug-Induced Reduction in 80Q Aggregates in a Dictyostelium discoideum Model of PolyQ Disease
Source: Biomedicines. 2026 Aug 28;14(9):1931. doi: 10.3390/biomedicines14091931 (PMC13604051; doi:10.3390/biomedicines14091931)
Supplement: Supplementary file 1 [file biomedicines-14-01931-s001.zip › biomedicines-4342864-supplementary.pdf]

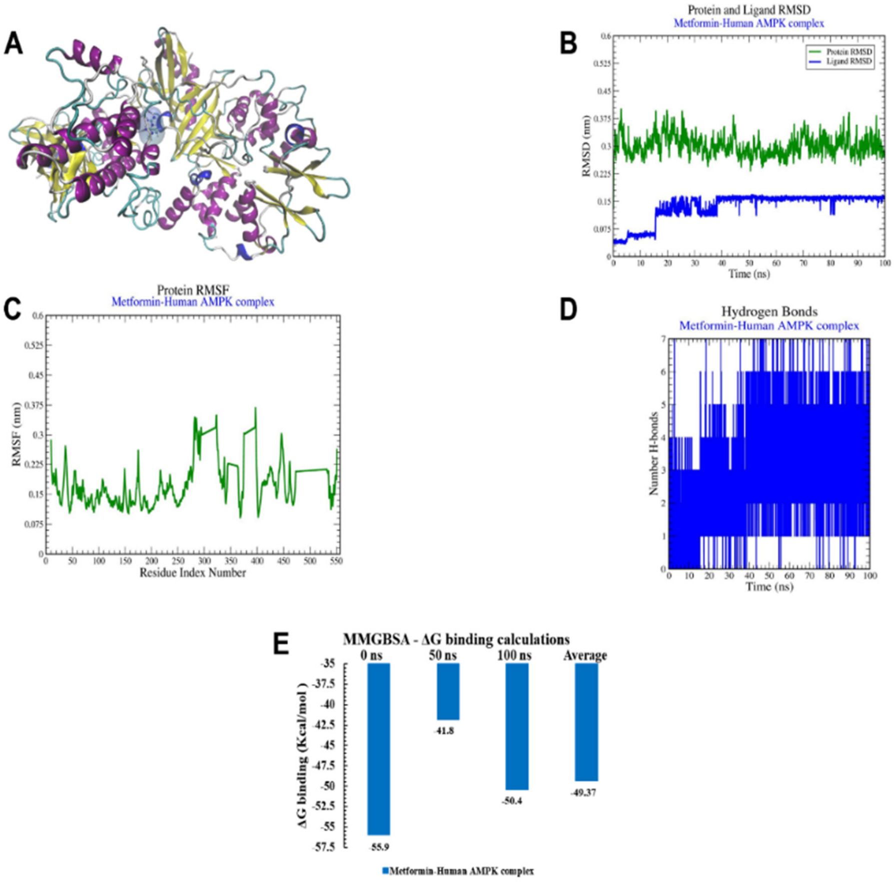

Supplementary Figure S1. Molecular Dynamics Analysis of the Metformin–Human AMPK Complex Reveals Stable Binding and Favorable Energetics

Sr.	Ligand	PIPER pose Score
1.	AMPK-ULK1 complex	-146.579

Supplementary Table S1. Docking summary for APK-ULK1 complex. Columns list the ligand or complex name, the top PIPER docking pose score (lower values indicate more favorable predicted binding). The top pose for the APK-ULK1 complex had a PIPER score of −146.579, no hydrogen bonds, and a pi-stack with Phe96.

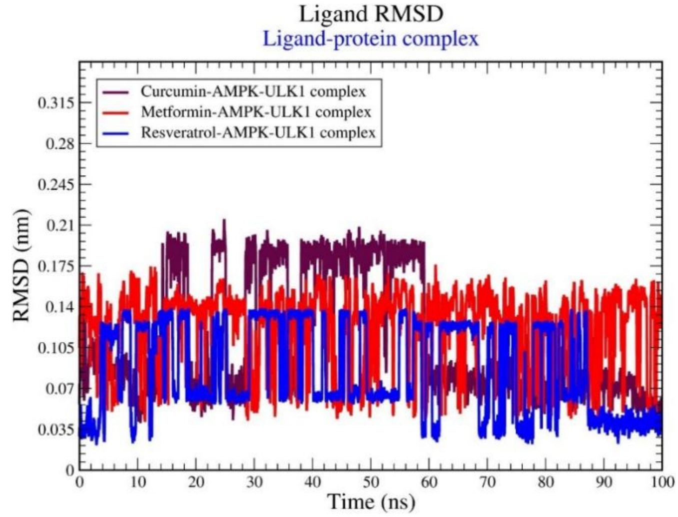

Supplementary Figure S2. RMSD analysis of ligand bound to the AMPK -ULK1 Complex over 100ns of Molecular dynamics simulations.The trajectories of curcumin, metformin and resveratrol were monitored to evaluate ligand positional stability within the binding pocket.All ligand exhibited low RMSD value, indicating stable binding thought out the simulation,with Resveratrol showing the most consistent behaviour and curcumin display slightly greater fluctuations during the mid simulation period

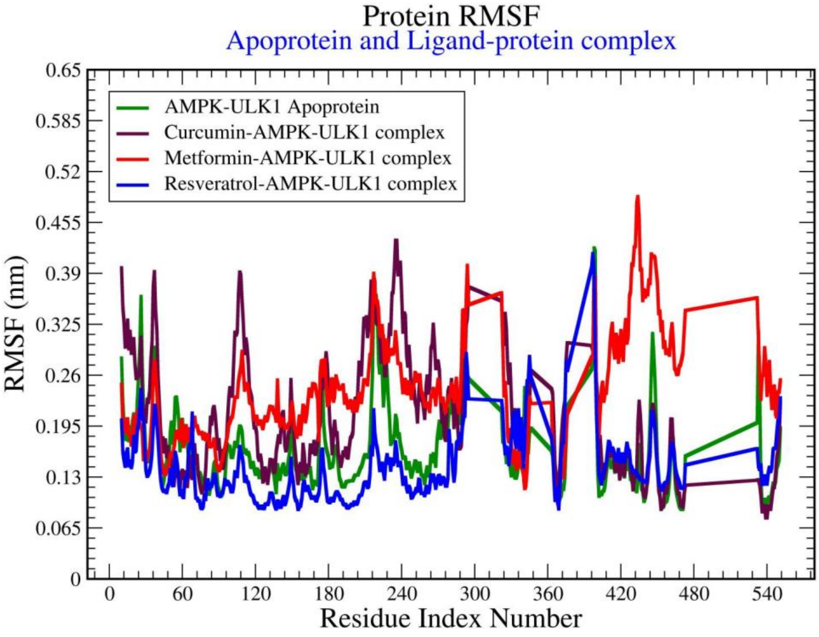

Supplementary Figure S3. Root mean square fluctuation (RMSF) analysis of the AMPK–ULK1 apoprotein and ligand-bound complexes. The graph illustrates the flexibility of each residue throughout the 100 ns

simulation for the apoprotein (green), curcumin (purple), metformin (red), and resveratrol (blue) systems. Variations in RMSF values highlight specific regions of structural mobility, with the resveratrol-bound complex demonstrating overall lower fluctuations, indicative of enhanced structural stability compared to the other complexes and the unbound apoprotein.

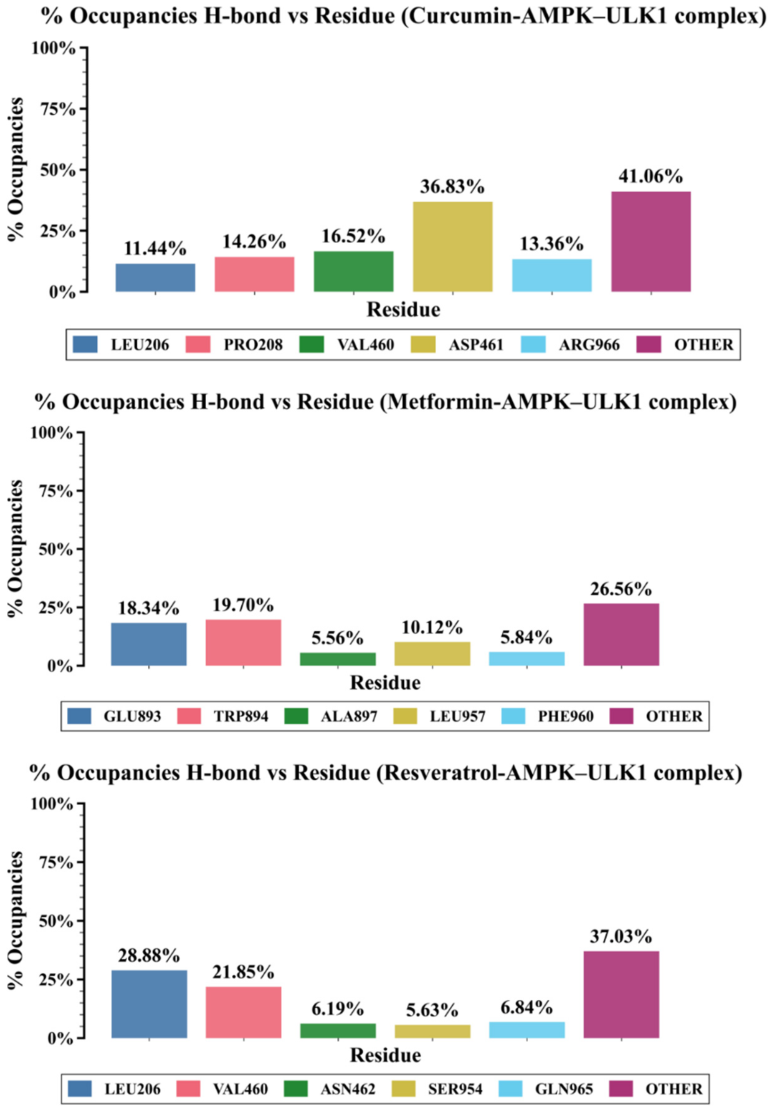

Supplementary Figure S4. Percentage hydrogen bond occupancy of key amino acid residues in the Metformin, Resveratrol, and Curcumin, AMPK–ULK1 complexes during molecular dynamics simulations. The occupancy values represent the persistence of hydrogen-bond interactions throughout the simulation trajectory. Curcumin exhibited the highest occupancy with ASP461 (36.83%), resveratrol showed strong interactions with LEU206 (28.88%) and VAL460 (21.85%), whereas metformin primarily interacted with TRP894 (19.70%) and GLU893 (18.34%). Higher occupancy percentages

indicate more stable and persistent ligand–protein interactions, highlighting the superior binding stability of curcumin and resveratrol compared with metformin.

Sr.	Complex	Mean_ΔG	SD	ΔE_vdW	ΔE_Elec	ΔE_Polar
1	AMPK–ULK1 Apoprotein	-27.15	3.11	-34.55	-7.35	19.98
2	Curcumin-AMPK–ULK1 complex	-15.50	2.51	-19.91	-2.69	10.28
3	Metformin-AMPK–ULK1 complex	-15.56	4.79	-19.55	-3.62	10.53

.

Supplementary Table S2. The result show that both curcumin and metformin complexes the strong negative contribution from van der waals interections effectively overcomes the destabilizing penalty of polar solvation, resulting in a net negative binding free energies that confirms stable, spontaneous complex formation.

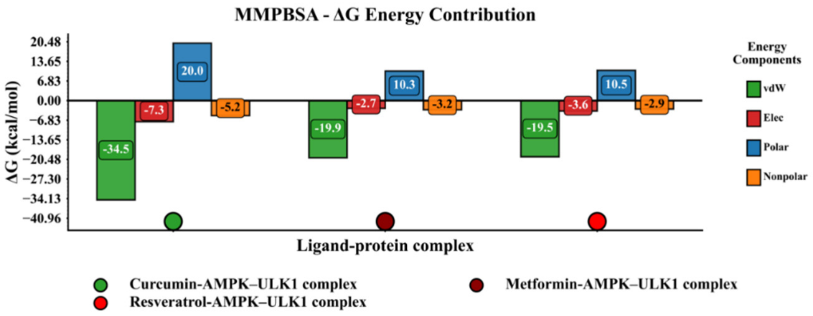

Supplementary Figure S5. Decomposition of binding free energies components for the AMPK -ULK1 - ligand system.the bar chart illustration the contribution of van der waals (green) , electrostatics (red),polar (blue),non polar (orange) energies to the total binding free energy.the predominance of negative van der waals values indicates that these interactions are the primary factors driving the structural stability and

binding affinity of the ligand - protein complex

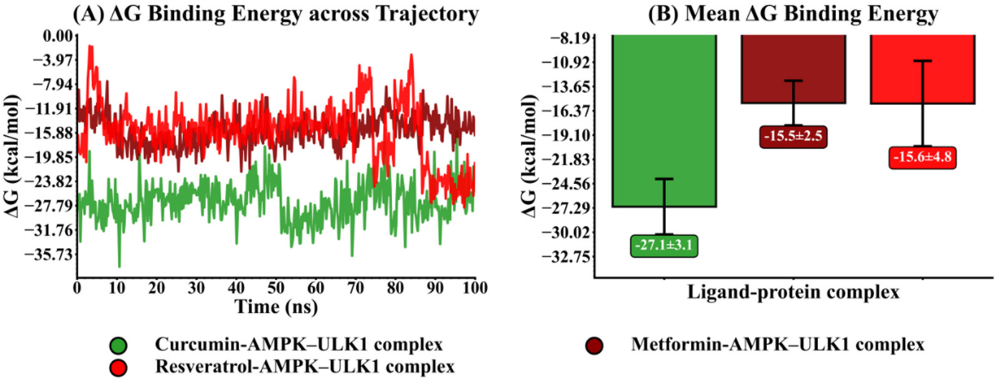

Supplementary Figure S6. Binding free energy (ΔG) profiles for ligand -protein complexes. (A)ΔG value calculated over the 100 ns simulation trajectory, demonstrating the time -dependent stability of the binding interaction.(B) Comparison of the mean binding free energy (± standard deviations) for each system, confirming the thermodynamic favorability and consistent binding affinity of the ligand within the AMPK -ULK1 binding pocket.

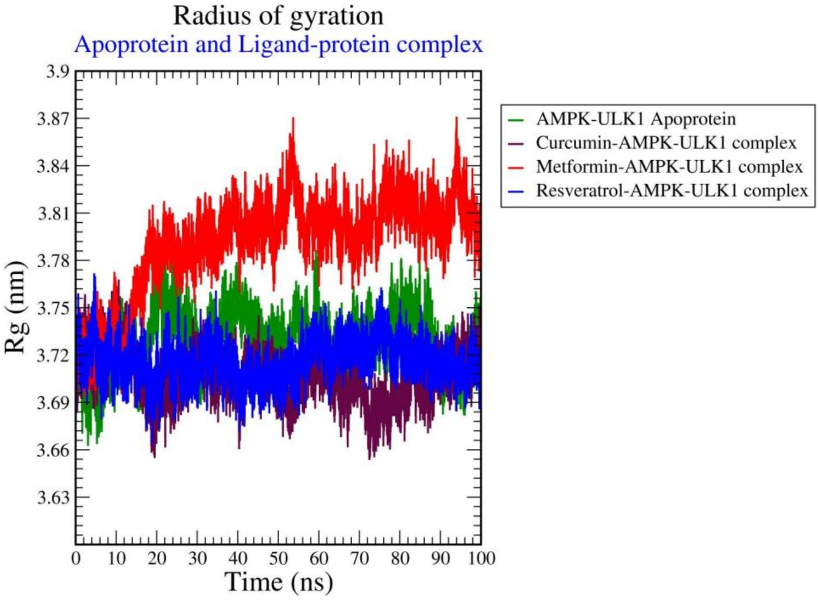

Supplementary Figure S7. Radius of gyration (R_g) analysis of the AMPK–ULK1 apoprotein and ligand-bound complexes over 100 ns of molecular dynamics simulation. The plot tracks the compactness of the protein structure for the apoprotein (green), curcumin (purple), metformin (red), and resveratrol (blue) systems. Consistent R_g values across the simulation indicate the maintenance of a stable global protein fold upon ligand binding, with resveratrol and curcumin complexes demonstrating enhanced structural compactness compared to the apoprotein and metformin systems.

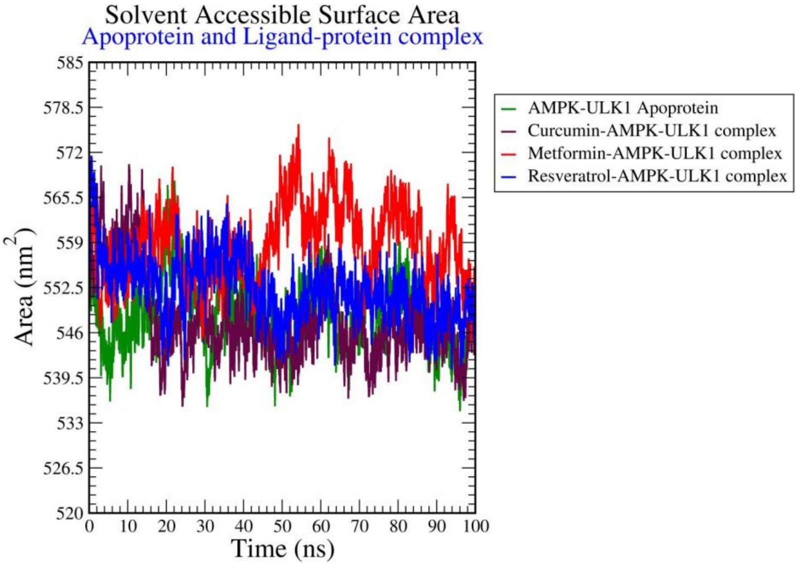

Supplementary Figure S8. Solvent accessible surface area (SASA) of the AMPK–ULK1 apoprotein and ligand-bound complexes during 100 ns molecular dynamics simulation. The SASA profiles of the apoprotein and its complexes with curcumin, metformin, and resveratrol are shown as a function of simulation time. The plot indicates that all systems remained relatively stable throughout the trajectory, with only minor fluctuations in solvent exposure.

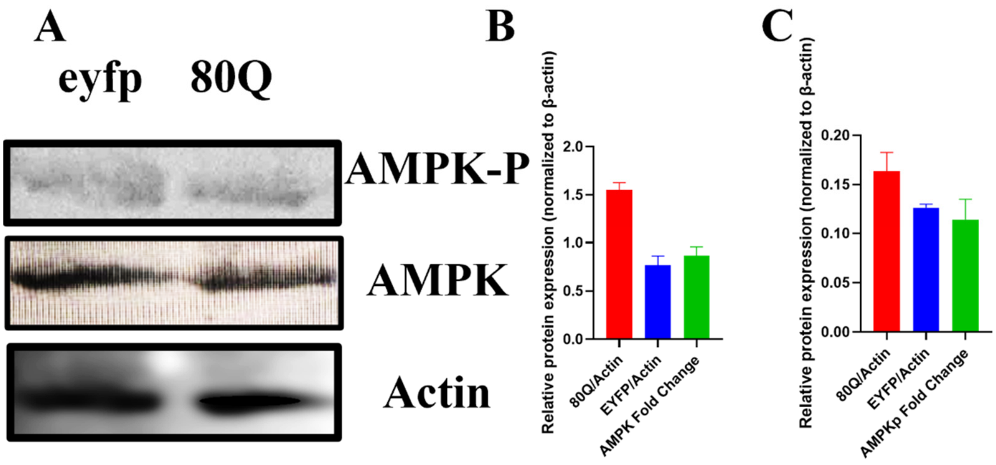

Supplementary Figure S9. (A) Representative Western blots showing β-actin, total AMPK, and phosphorylated AMPK (AMPK-P) protein levels in eYFP control and eYFP-80Q cells.**(B)** Quantification of total AMPK protein expression normalized to β-actin and expressed relative to the eYFP control.**(C)** Quantification of phosphorylated AMPK (AMPK-P) levels

normalized to β-actin and expressed relative to the eYFP control.Data are presented as mean ± SD from three independent experiments (n = 3).

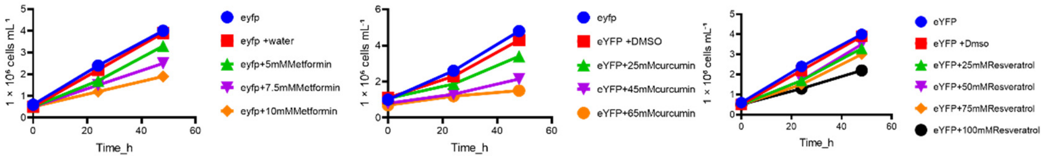

Supplementary Figure S10. Dose-response curves were generated using cell number measurements following treatment with increasing concentrations of metformin (5, 7.5, and 10 μM), curcumin (25, 45, and 65 μM), and resveratrol (25, 50, 75, and 100 μM). Data are presented as mean ± SD from three independent experiments (n = 3). IC_{50} values were calculated by nonlinear regression analysis using GraphPad Prism 8.0. Based on these analyses, 10 μM metformin, 45 μM curcumin, and 100 μM resveratrol were selected for subsequent experiments.

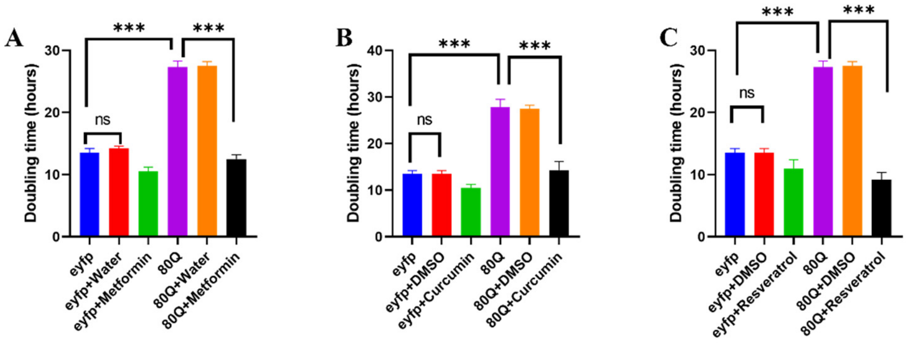

Supplementary Figure S11. (A) Metformin did not significantly alter the doubling time of eyfp control cells, whereas 80Q expression markedly increased it.
(B) Curcumin showed a similar pattern, with no significant effect on eyfp cells but a significant reduction in the elevated doubling time of 80Q cells.
(C) Resveratrol also significantly lowered the prolonged doubling time in 80Q-expressing cells.
Data are shown as mean ± SD, and *** indicates $P < 0.001$.

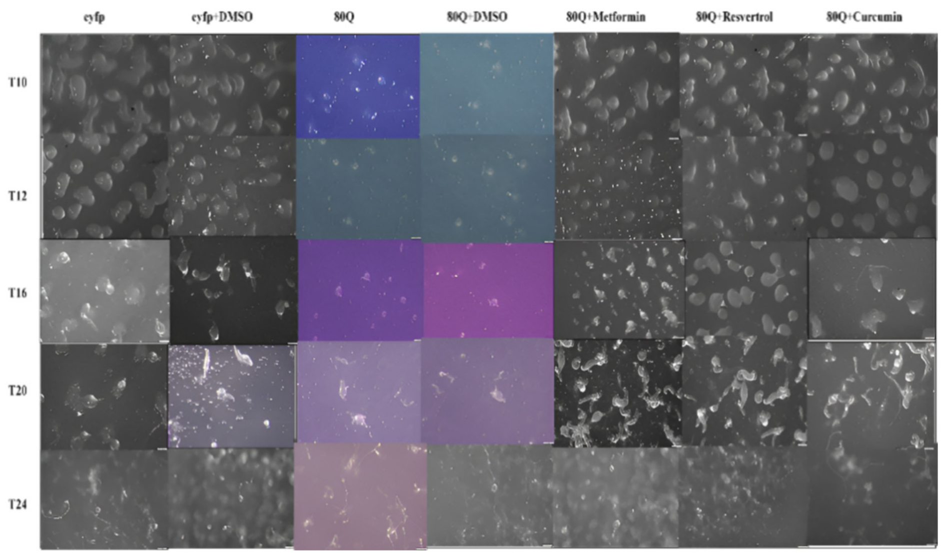

Supplementary Figure S12. multicellular development in cells expressing control or 80Q polyQ eYFP in the absence or presence of metformin, curcumin, or resveratrol. The expression of 80Q resulted in strongly suppressed cell numbers, size, and arrangement in developmental structures in terms of late developmental phenotypes. The addition of drugs enhanced cell numbers, size, and arrangement in both cell aggregates, slugs, and culminants; in addition to this, the cell structures were more elongated and properly orientated compared to drug-treated 80Q.

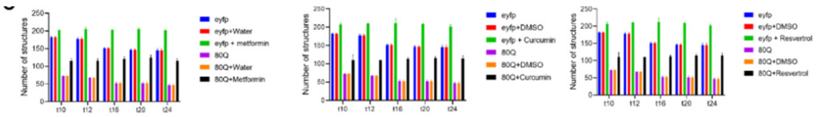

Supplementary Figure S13. Quantitative Analysis of Growth Rescue.Bar graphs representing quantitative comparison of cell number/growth in eYFP control cells, 80Q cells, and 80Q cells treated with metformin, curcumin, or resveratrol. The results reveal that the overexpression of 80Q markedly inhibited cell proliferation when compared to the control cells. However, metformin, curcumin, and resveratrol partially, but significantly, countered this phenomenon. Curcumin was more effective in reversing this condition than resveratrol.Data are presented as mean ± SD from three independent experiments (n = 3).

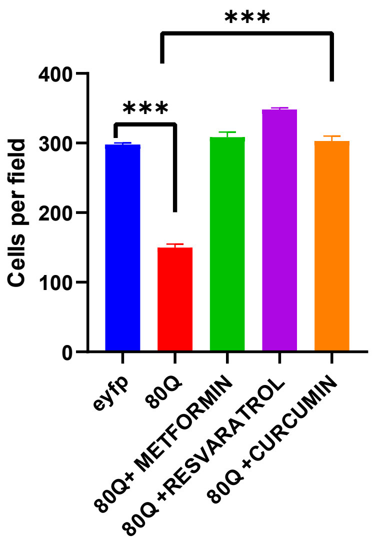

Supplementary Figure S14. Microscopy-based cell counting showed that 80Q expression reduced cell number to about half of eYFP controls, while metformin, resveratrol, and curcumin treatment restored or exceeded control cell numbers, demonstrating a robust protective effect on polyQ-induced growth defects.Data are presented as mean ± SD from three independent experiments (n = 3).

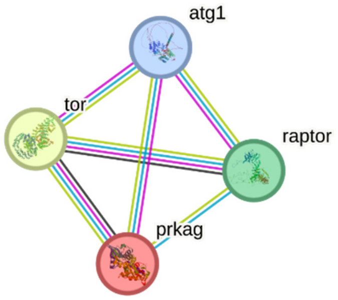

Supplementary Figure S15. STRING interaction network showing the functional associations between autophagy- and AMPK-related proteins (ATG1, TOR, RAPTOR, and PRKAG). The dense interactions among these proteins indicate their cooperative involvement in regulating autophagy, metabolic signaling, and neuroprotective mechanisms.
